# Supplementary material for: Early and adult life environmental effects on reproductive performance in preindustrial women
Source: PLoS One. 2024 Oct 28;19(10):e0290212. doi: 10.1371/journal.pone.0290212 (PMC11515999; doi:10.1371/journal.pone.0290212)
Supplement: S1 Appendix — (DOCX) [file pone.0290212.s001.docx]

# Additional analyses

We performed additional analysis on *fertile years* and the Proportion between LRS and NO (LRS/NO). The model for *fertile years* was normally distributed and analysed with a linear mixed model (LMM). Although *fertile years* and *wave front* are not correlated (r = -0.08, p <0.001, N=7,203), it’s important to consider that both variables represent a number of years during a specific period. This means that, when interpreting the results, it’s crucial to recognise that they could capture some of the same variance in the model. The LRS/NO was analysed using a GLMM, with a beta-binomial distribution, to account for overdispersion in the data (dispersion parameter for beta-binomial family = 25.3).

## Environmental effects on fertile years

The model explaining the fertile years indicated as significant the environmental switch between birth and adulthood (*Switching Urbanity*: χ² [2] = 18.54, p <0.001)*,* the *wave front* (S4 Table), and the *period* (χ² [4] = 51.90, p <0.001). The early life environment (*Birth Environment*: χ² [3] = 6.40, p = 0.009) and the switch of shore (*Switching Shore*: χ² [2] = 2.57, p =0.276) were not significant. For the switch in urbanity, individuals who stayed under the same conditions had 1.1 fertile years more than individuals who moved from rural to urban (good to bad), which was significant according to the Tukey test (S4B Fig). Most of the variance of the model was explained by the *wave front*, followed by the *period,* and the *switch in urbanity* (S5 Table). Overall, the model for fertile years only explained 2% of the variance with fixed effects.

## Environmental effects on the Proportion Between LRS and NO

The model explaining the proportion between LRS and NO included considered as significant the early life environment (*Birth Environment*: χ² [3] = 44.03, p <0.001) and the environmental switch before adulthood (*Switching Urbanity* Switching Shore*: χ² [4] = 9.80, p <0.001) as well as *fertile years* and *wave front* (S4 Table). For the early life environment, women born in urban parishes had a proportion of offspring surviving to adulthood 0.65 points lower on average than the ones born in rural parishes (S3C Fig), which was significant according to the Tukey test. However, there was no significant difference between women born on the south shore compared to the north shore, as indicated by the Tukey test (S3C Fig). For the environmental switches before adulthood (S4E Fig), women who moved from rural to urban areas (good to bad) had offspring survival rates to adulthood that were 0.55-0.86 points lower than those who remained in the same conditions and 1.03-1.55 points lower than those who moved from urban to rural areas (bad to good), depending on the shore switch. These differences were significant according to the Tukey test (S4E Fig). Therefore, women who transitioned from urban to rural environments (bad to good) had offspring survival rates to adulthood that were 0.5-1.0 points higher, depending on the shore switch, compared to those who remained under the same conditions. These differences were statistically significant according to the Tukey test (S4E Fig). The distance between the early and adult life environments was negatively related to the proportion between LRS and NO (S4 Table and S6B Fig). We could not analyse the partitioned R^2^ of the final model, since it is not possible to do so when studying proportions.
