# Supplementary material for: Early and adult life environmental effects on reproductive performance in preindustrial women
Source: PLoS One. 2024 Oct 28;19(10):e0290212. doi: 10.1371/journal.pone.0290212 (PMC11515999; doi:10.1371/journal.pone.0290212)
Supplement: S2 Appendix — (DOCX) [file pone.0290212.s002.docx]

# Sensitivity Analysis

We performed a sensitivity analysis on the Age at Marriage to ensure the accuracy of the measurements for Age at First Reproduction. Age at marriage and Age at First Reproduction are highly correlated (r = 0.95, p <0.001) in this population (N=7,203), since the first birth occurred on average 1.2 years after the wedding. Age at Marriage was normally distributed and analysed with a linear mixed model (LMM).

## Environmental Effects on the Age at Marriage

The model explaining Age at Marriage deemed as significant both early life environment (LRT *Birth Environment*: χ² [3] = 106.25, p <0.001) and environmental switching between birth and adulthood (LRT *Switching Urbanity* * *Switching Shore*: χ² [4] = 4.42, p= 0.352), as well as the *wave front*, *distance,* and *period* (Table S4). Compared to women born in rural areas, those born in urban areas got married on average 3.4 years earlier if born south and 4.7 years if born north (S5A Fig), and this difference was significant according to the Tukey test. In addition, women born in the southern urban parishes got married 1.3 years later than those born in the northern urban ones, which was also significant according to the Tukey test (S5A Fig). For the interaction between *Switching Urbanity* and *Switching Shore*, women who switched from rural to urban and south to north married 1.4 years later than those who remained in the same urbanity and same shore and 1.3 years later than the ones who remained in the same urbanity and switched from north to south, which were the only combinations that the Tukey test indicated as significantly different (S5B Fig). *Distance* between early and adult life environments also influenced Age at Marriage (S4 Table); women who moved greater distances married later (S6C Fig). Overall, 13% of the variance in Age at Marriage was explained by the fixed effects of the final model.
