# Supplementary material for: Early and adult life environmental effects on reproductive performance in preindustrial women
Source: PLoS One. 2024 Oct 28;19(10):e0290212. doi: 10.1371/journal.pone.0290212 (PMC11515999; doi:10.1371/journal.pone.0290212)
Supplement: S3 Appendix — (DOCX) [file pone.0290212.s003.docx]

# References of the Supporting Information

S1. Données Québec. (2019). Base de données géographiques et administratives. https://www.donneesquebec.ca/recherche/dataset/base-de-donnees-geographiques-et-administratives/resource/ee928e60-5fef-488d-af37-099281a4e0e6
