# Supplementary figures and images for: Early and adult life environmental effects on reproductive performance in preindustrial women
Source: PLoS One. 2024 Oct 28;19(10):e0290212. doi: 10.1371/journal.pone.0290212 (PMC11515999; doi:10.1371/journal.pone.0290212)

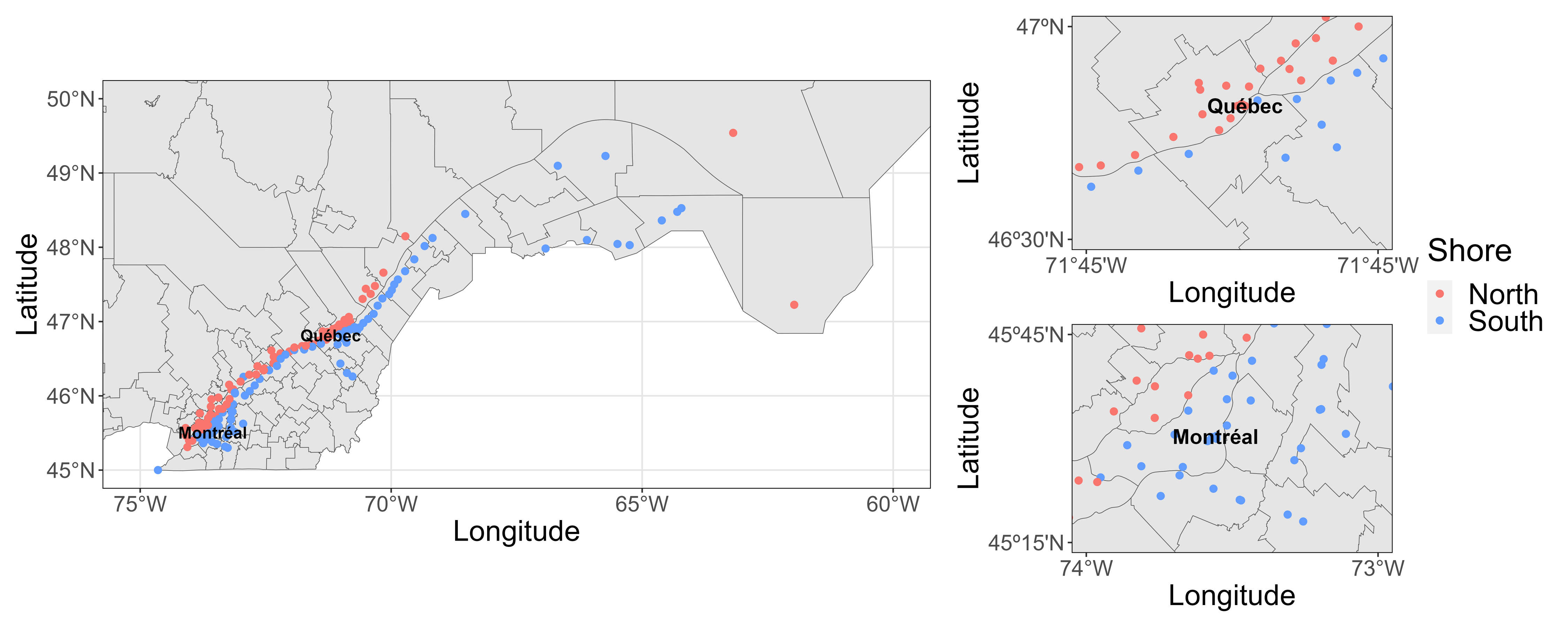

Supplement: S1 Fig — Panel A shows the location of the parishes along the St. Lawrence Valley, panel B shows a zoom into the area near Québec City and panel C shows a zoom into the area near Montréal. Conditions in the rural parishes were different from those in Québec City and Montréal, and conditions on the north shore (points in red) were also different from those in the south (points in blue). Conditions in the two cities were also different from each other. Modified from “Base de données géographiques et administratives, Données Québec”, under a CC BY 4.0 license, with permission from Données Québec, original copyright 2019. (TIF) [file pone.0290212.s004.tif]

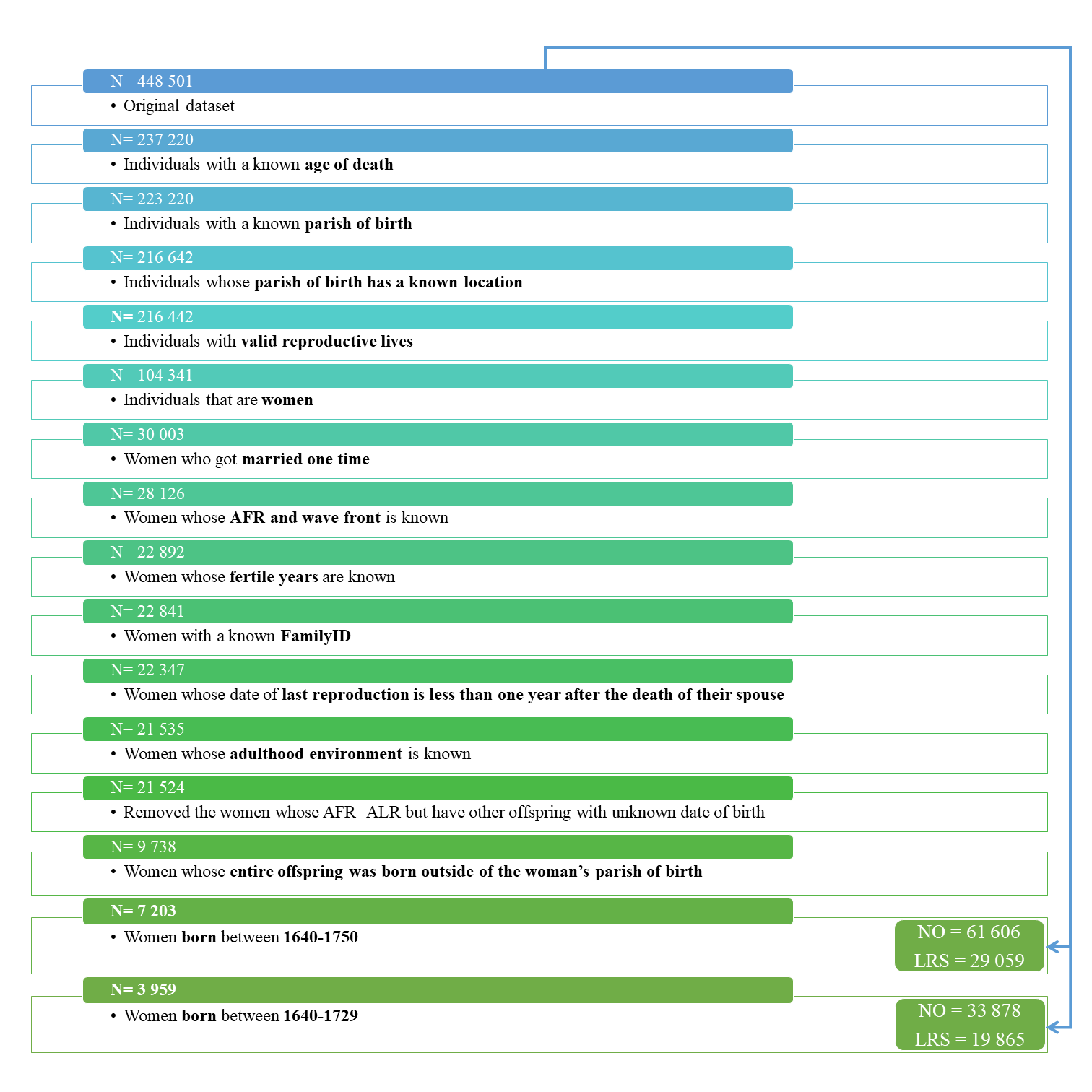

Supplement: S2 Fig — It also indicates which subset was used to calculate the number of offspring (NO) and lifetime reproductive success (LRS). (TIF) [file pone.0290212.s005.tif]

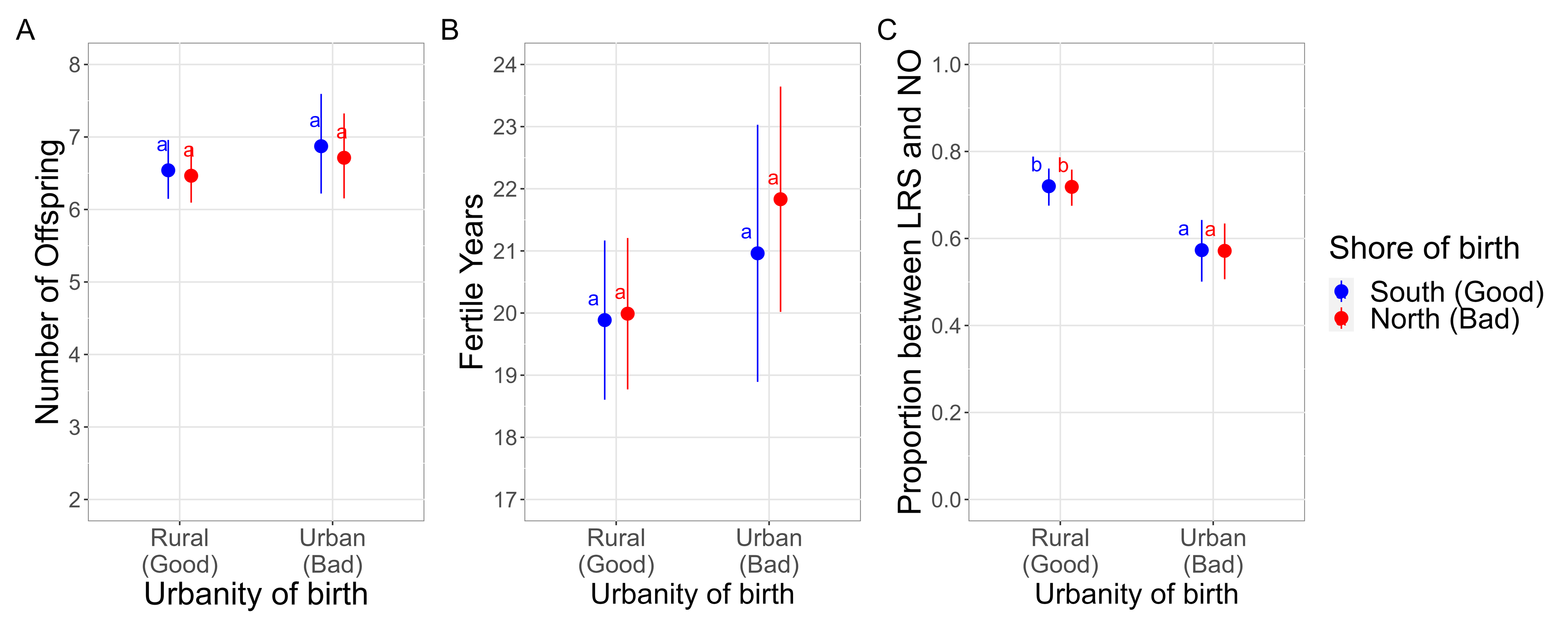

Supplement: S3 Fig — Early life environmental effects on the Number of Offspring (Panel A), Fertile Years (Panel B) and the Proportion between LRS and NO (panel C) according to the environment of birth, given by the urbanity (Rural or Urban) and the shore (North and South). Rural and South are considered good environments and Urban and North are considered bad environments. The dots are the predicted marginal values, and the lines are their confidence intervals. Estimates with different letters are statistically different, given by a post-hoc Tukey’s pairwise multiple comparison test (P <0.05). (TIF) [file pone.0290212.s006.tif]

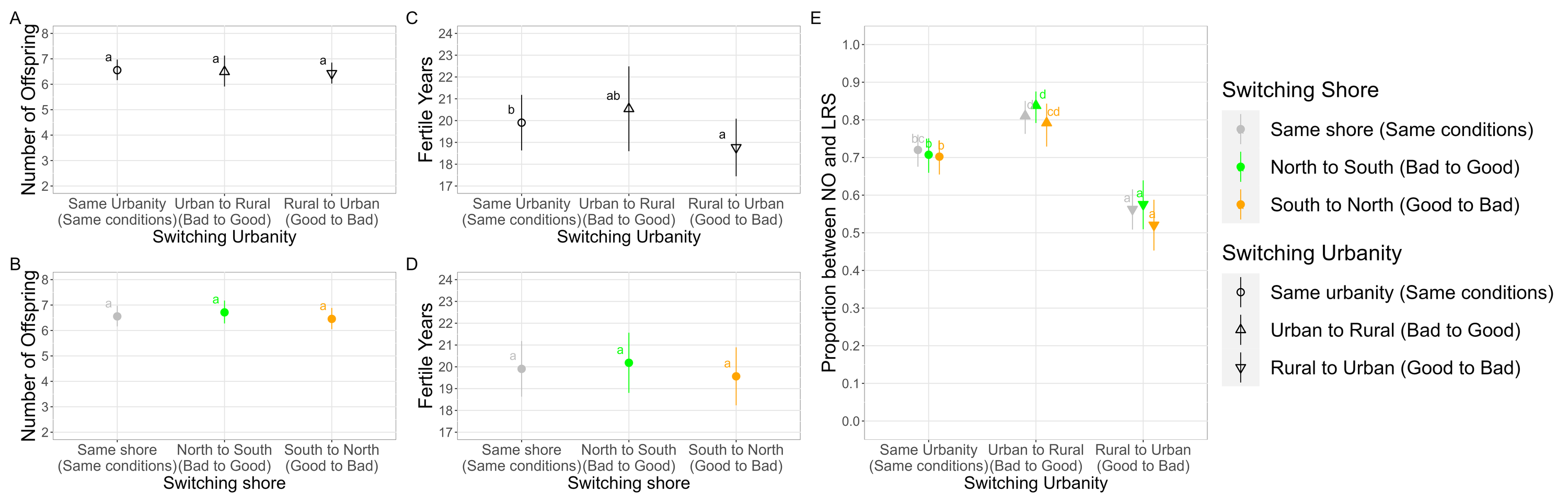

Supplement: S4 Fig — Adult life environmental effects on the Number of Offspring (Panels A and B), Fertile years (Panels C and D) and the Proportion between LRS and NO (Panel E) according to the switch in urbanity, the switch in shore, and the interaction between them. Staying in the same urbanity or in the same shore is considered as staying under the same conditions, while switching from urban to rural or north to south is seen as going from a bad to a good environment, and switching from rural to urban or south to north is considered going from a good to a bad environment. The dots are the predicted marginal values, and the lines are their confidence intervals. Estimates with different letters are statistically different, given by a post-hoc Tukey’s pairwise multiple comparison test (P <0.05). (TIF) [file pone.0290212.s007.tif]

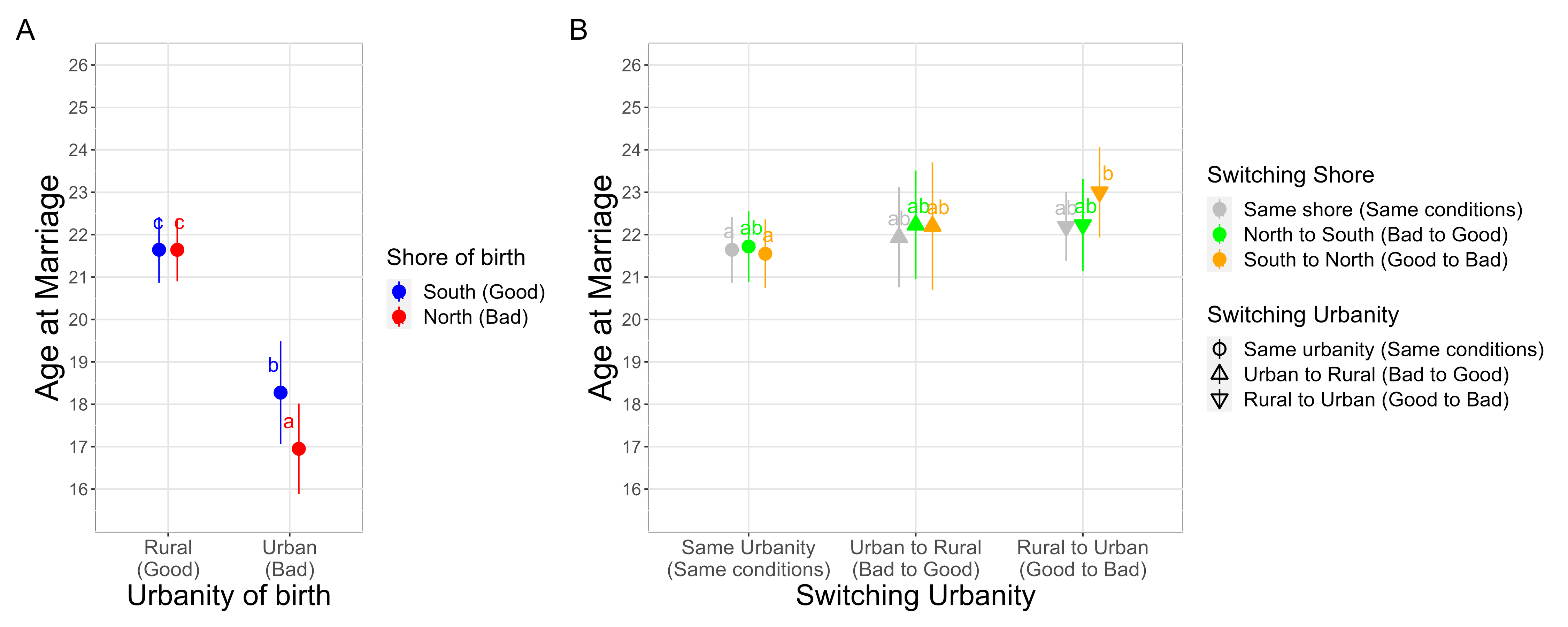

Supplement: S5 Fig — Early life environmental effects (Panel A) according to the environment of birth, given by the urbanity (Rural or Urban) and the shore (North and South) and adult life environmental effects (Panel B), according to the interaction between the switch in urbanity and the switch in shore, on Age at Marriage. Rural and South are considered good environments and Urban and North are considered bad environments. Staying in the same urbanity or in the same shore is considered as staying under the same conditions, while switching from urban to rural or north to south is seen as going from a bad to a good environment, and switching from rural to urban or south to north is considered going from a good to a bad environment. The dots are the predicted marginal values, and the lines are their confidence intervals. Estimates with different letters are statistically different, given by a post-hoc Tukey’s pairwise multiple comparison test (P <0.05). (TIF) [file pone.0290212.s008.tif]

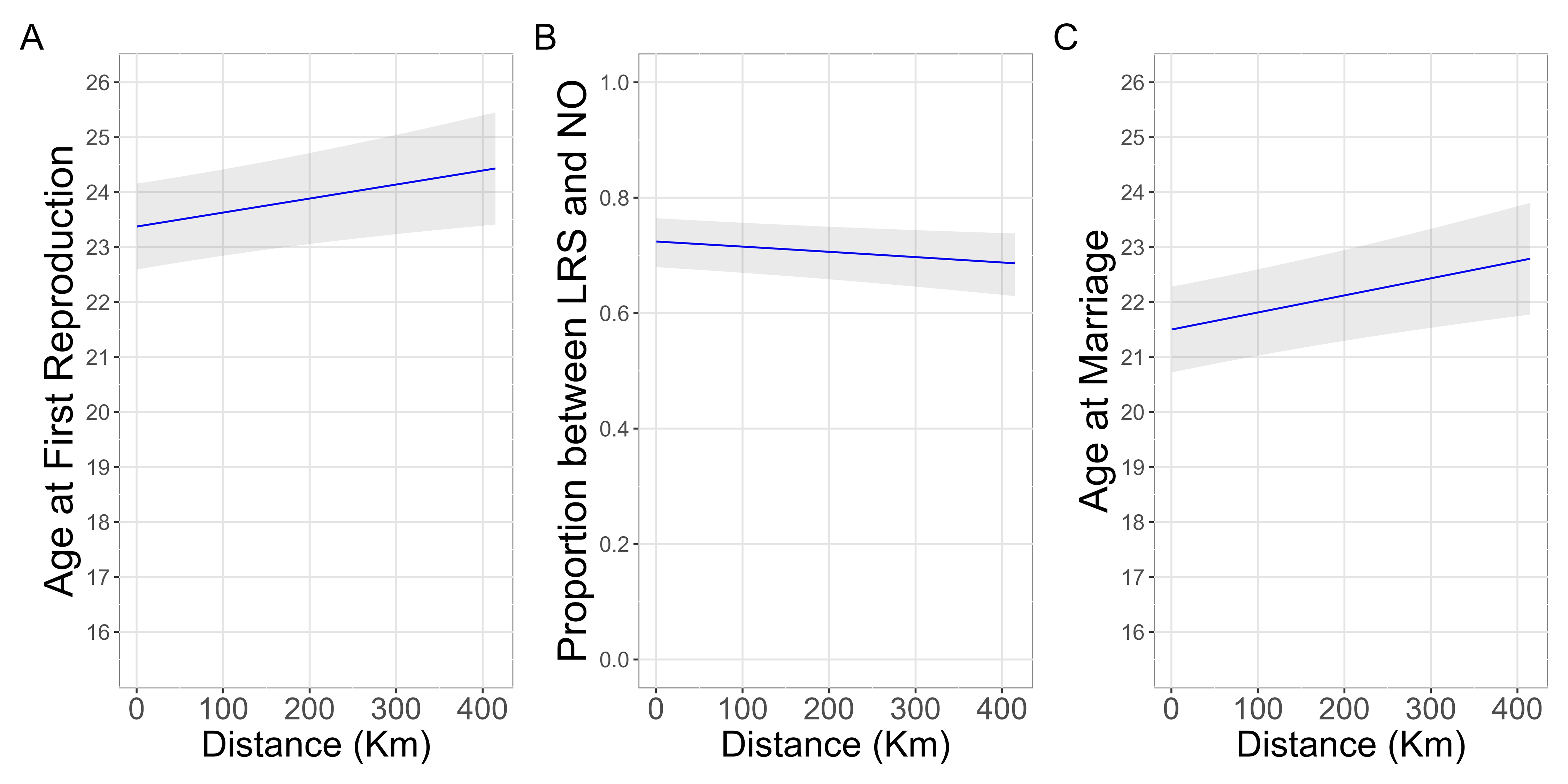

Supplement: S6 Fig — Effects of the distance between the parish of birth and the parish of first reproduction on reproductive performance, given by Age at First Reproduction (Panel A), the Proportion between LRS and NO (Panel B) the Age at Marriage (Panel C). The blue lines are the predicted marginal values, and the shaded areas describe the confidence interval. (TIF) [file pone.0290212.s009.tif]

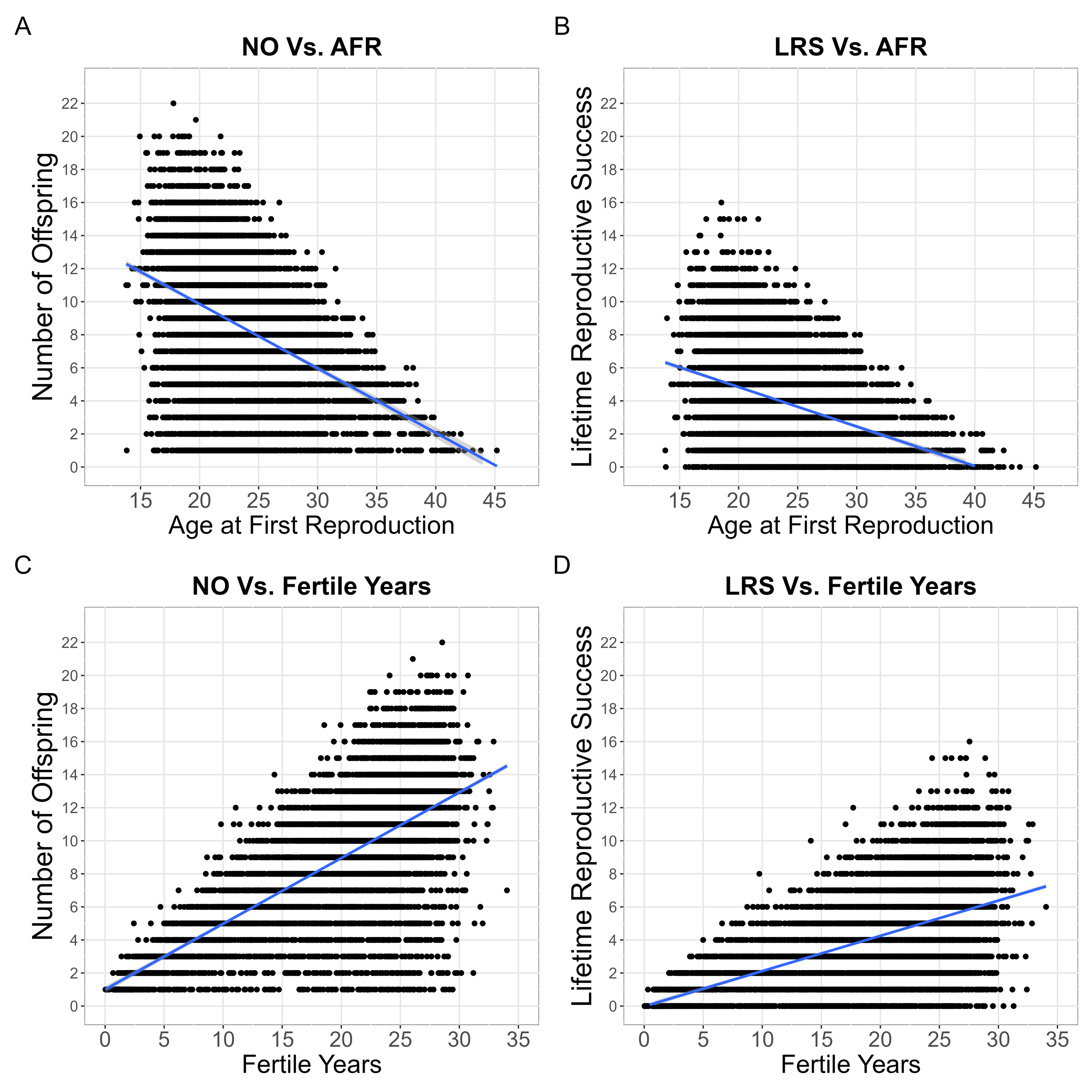

Supplement: S7 Fig — Panel A shows the relationship between the age at first reproduction (AFR) in years and the number of offspring (NO) in terms of children born. Panel B displays the correlation between AFR and lifetime reproductive success (LRS) in terms of the number of children who survived to adult life. Panel C illustrates the correlation between the number of offspring (NO) and the fertile years in years. Finally, Panel D exhibits the correlation between the fertile years and lifetime reproductive success in terms of the number of children who survived to adult life. (TIF) [file pone.0290212.s010.tif]
