# Supplementary material for: Early and adult life environmental effects on reproductive performance in preindustrial women
Source: PLoS One. 2024 Oct 28;19(10):e0290212. doi: 10.1371/journal.pone.0290212 (PMC11515999; doi:10.1371/journal.pone.0290212)
Supplement: S1 Table — They are calculated for N = 7,203, except Lifetime Reproductive Success which is calculated for N = 3,959. (DOCX) [file pone.0290212.s011.docx]

**S1 Table. Descriptive statistics of the means of all the reproductive traits analysed.** They are calculated for N= 7,203, except Lifetime Reproductive Success which is calculated for N= 3,959.

|  | *Age at Marriage* | *Age at First Reproduction* | *Number of Offspring* | *Lifetime Reproductive Success* | *Fertile Years* |
| --- | --- | --- | --- | --- | --- |
| *Rural* | 22.32 | 23.51 | 8.50 | 4.95 | 18.88 |
| *Urban* | 21.20 | 22.43 | 8.85 | 5.34 | 19.68 |
| *South* | 21.97 | 23.18 | 8.62 | 4.91 | 18.91 |
| *North* | 22.33 | 23.52 | 8.49 | 5.10 | 19.07 |
| *Rural South* | 22.07 | 23.29 | 8.57 | 4.87 | 18.84 |
| *Rural North* | 22.55 | 23.72 | 8.44 | 5.01 | 18.92 |
| *Urban South* | 21.25 | 22.44 | 9.01 | 5.14 | 19.42 |
| *Urban North* | 21.16 | 22.42 | 8.73 | 5.50 | 19.87 |
| *Total* | **22.16** | **23.36** | **8.55** | **5.02** | **18.99** |
