# Supplementary material for: Early and adult life environmental effects on reproductive performance in preindustrial women
Source: PLoS One. 2024 Oct 28;19(10):e0290212. doi: 10.1371/journal.pone.0290212 (PMC11515999; doi:10.1371/journal.pone.0290212)
Supplement: S2 Table — (DOCX) [file pone.0290212.s012.docx]

**S2 Table. Descriptive statistics on the distribution of the population according to the environment of birth, for N= 7,203.**

|  | *Rural* | | *Urban* | | *Total* | |
| --- | --- | --- | --- | --- | --- | --- |
|  | *N* | *%* | *N* | *%* | *N* | *%* |
| *South* | 3009 | *41.8* | 439 | *6.1* | 3448 | *47.9* |
| *North* | 3171 | *44.0* | 584 | *8.1* | 3755 | *52.1* |
| *Total* | 6180 | *85.8* | 1023 | *14.2* | 7203 | *100* |
