# Supplementary material for: Early and adult life environmental effects on reproductive performance in preindustrial women
Source: PLoS One. 2024 Oct 28;19(10):e0290212. doi: 10.1371/journal.pone.0290212 (PMC11515999; doi:10.1371/journal.pone.0290212)
Supplement: S3 Table — (DOCX) [file pone.0290212.s013.docx]

**S3 Table. Descriptive statistics on the distribution of the population according to the environmental switch, for N= 7,203.**

|  | *Same conditions* | | *Rural to Urban* | | *Urban to Rural* | | *Total* | |
| --- | --- | --- | --- | --- | --- | --- | --- | --- |
|  | *N* | *%* | *N* | *%* | *N* | *%* | *N* | *%* |
| *Same conditions* | 3594 | *49.9* | 624 | *8.7* | 671 | *9.3* | 4889 | *67.9* |
| *South to North* | 1119 | *15.5* | 193 | *2.7* | 129 | *1.8* | 1441 | *20.0* |
| *North to South* | 669 | *9.3* | 88 | *1.2* | 116 | *1.6* | 873 | *12.1* |
| *Total* | 5382 | *74.7* | 905 | *12.6* | 916 | *12.7* | 7203 | *100* |
