# Supplementary material for: Early and adult life environmental effects on reproductive performance in preindustrial women
Source: PLoS One. 2024 Oct 28;19(10):e0290212. doi: 10.1371/journal.pone.0290212 (PMC11515999; doi:10.1371/journal.pone.0290212)
Supplement: S4 Table — (DOCX) [file pone.0290212.s014.docx]

**S4 Table. Output of the full models with all the variables present in the first column, for Fertile Years, the Proportion between Lifetime Reproductive Success and Number of Offspring (LRS/NO) and the Age at Marriage.**

| *Variables* | | *Fertile Years* | | | *LRS/NO* | | | *Age at Marriage* | | | |
| --- | --- | --- | --- | --- | --- | --- | --- | --- | --- | --- | --- |
|  |  | Estimate | SE | P value | Estimate | SE | P value | Estimate | SE | P value |  |
| *(Intercept)* | | 19.887 | 0.654 | ***< 0.001*** | 0.945 | 0.108 | ***< 0.001*** | 21.643 | 0.397 | ***< 0.001*** |  |
| *Birth Environment* | *Rural North* | 0.103 | 0.235 | *0.661* | -0.007 | 0.029 | *0.798* | -0.002 | 0.139 | *0.989* |  |
|  | *Urban South* | 1.075 | 0.908 | *0.236* | -0.650 | 0.112 | ***< 0.001*** | -3.369 | 0.523 | ***< 0.001*** |  |
|  | *Urban North* | 1.945 | 0.840 | ***0.021*** | -0.657 | 0.105 | ***< 0.001*** | -4.693 | 0.486 | ***< 0.001*** |  |
| *Fertile years* | | *—* | *—* | *—* | 0.142 | 0.013 | ***< 0.001*** | *—* | *—* | *—* |  |
| *Wave front* | | -1.166 | 0.137 | ***<0.001*** | -0.027 | 0.018 | *0.132* | 1.850 | 0.081 | ***<0.001*** |  |
| *Distance* | | 0.042 | 0.100 | *0.672* | -0.029 | 0.012 | ***0.020*** | 0.203 | 0.058 | ***<0.001*** |  |
| *Period* | *1670–1689* | -1.283 | 0.615 | ***0.037*** | -0.103 | 0.121 | *0.393* | 1.891 | 0.380 | ***<0.001*** |  |
|  | *1690–1709* | -1.624 | 0.614 | ***0.008*** | -0.306 | 0.121 | ***0.011*** | 2.233 | 0.383 | ***<0.001*** |  |
|  | *1710–1729* | -2.000 | 0.627 | ***0.001*** | -0.743 | 0.122 | ***<0.001*** | 1.507 | 0.391 | ***<0.001*** |  |
|  | *1730–1750* | -0.475 | 0.648 | *0.463* | -1.583 | 0.123 | ***<0.001*** | 0.057 | 0.402 | *0.888* |  |
| *Switching Urbanity* | *Urban to Rural* | 0.772 | 0.835 | *0.356* | 0.507 | 0.103 | ***<0.001*** | 0.294 | 0.478 | *0.538* |  |
|  | *Rural to Urban* | -1.206 | 0.315 | ***<0.001*** | -0.693 | 0.040 | ***<0.001*** | 0.548 | 0.181 | ***0.003*** |  |
| *Switching Shore* | *North to South* | 0.291 | 0.291 | *0.318* | -0.063 | 0.035 | *0.076* | 0.077 | 0.168 | *0.649* |  |
|  | *South to North* | -0.346 | 0.330 | *0.294* | -0.087 | 0.040 | ***0.030*** | -0.094 | 0.190 | *0.621* |  |
| *Switching Urbanity*  ***  *Switching Shore* | *Urban to Rural.*  *North to South* | -0.426 | 0.735 | *0.562* | 0.252 | 0.090 | ***0.005*** | 0.216 | 0.422 | *0.609* |  |
|  | *Rural to Urban.*  *North to South* | 0.432 | 0.767 | *0.574* | 0.115 | 0.095 | *0.227* | -0.038 | 0.443 | *0.931* |  |
|  | *Urban to Rural.*  *South to North* | -0.021 | 0.932 | *0.982* | -0.030 | 0.113 | *0.791* | 0.358 | 0.530 | *0.500* |  |
|  | *Rural to Urban.*  *South to North* | 0.084 | 0.808 | *0.917* | -0.083 | 0.105 | *0.430* | 0.906 | 0.462 | ***0.050*** |  |

*The reference level is “Rural South” for “Birth Environment”, “Same Urbanity” for “Switching Urbanity”, “Same Shore” for “Switching Shore” and ’1640–1669’ for “Period”. The variable “fertile years” was not included in the AFR model as fixed effect. “—” means that the variable was not included in the full model. P values in bold are significative (p <0.05).*
