# Supplementary material for: Early and adult life environmental effects on reproductive performance in preindustrial women
Source: PLoS One. 2024 Oct 28;19(10):e0290212. doi: 10.1371/journal.pone.0290212 (PMC11515999; doi:10.1371/journal.pone.0290212)
Supplement: S5 Table — These contributions were calculated by hierarchically partitioning the marginal R² of the generalized mixed-effect model, based on the output from the r.squaredGLMM() function in the “MuMIn” package. (DOCX) [file pone.0290212.s015.docx]

**S5 Table. Contributions of each fixed effect for each full model were determined by using the “glmm.hp” package. These contributions were calculated by hierarchically partitioning the marginal R² of the generalized mixed-effect model, based on the output from the r.squaredGLMM() function in the “MuMIn” package.**

|  | *AFR* | | | | *NO* | | | | | *LRS* | | | | | *Fertile Years* | | | | |  |
| --- | --- | --- | --- | --- | --- | --- | --- | --- | --- | --- | --- | --- | --- | --- | --- | --- | --- | --- | --- | --- |
|  | *Uniq.* | *Avg. share* | *Indiv.* | *I. perc*  *(%)* | | *Uniq.* | *Avg. share* | *Indiv.* | *I. perc*  *(%)* | | *Uniq.* | *Avg. share* | *Indiv.* | *I. perc*  *(%)* | | *Uniq.* | *Avg. share* | *Indiv.* | *I. perc*  *(%)* | |
| *Birth Environment* | 0.016 | 0.005 | 0.021 | *16.61* | | 0.000 | 0.002 | 0.002 | *0.26* | | 0.003 | 0.003 | 0.006 | *1.11* | | 0.001 | 0.002 | 0.002 | *8.98* | |
| *Wave front* | 0.078 | -0.002 | 0.076 | *61.29* | | 0.000 | 0.007 | 0.007 | *1.17* | | 0.000 | 0.020 | 0.021 | *3.74* | | 0.010 | 0.000 | 0.010 | *40.62* | |
| *Fertile years* | — | — | — | — | | 0.544 | 0.012 | 0.556 | *97.46* | | 0.391 | 0.051 | 0.442 | *80.64* | | — | — | — | — | |
| *Distance* | 0.001 | 0.000 | 0.002 | *1.37* | | 0.000 | 0.000 | 0.000 | *0.00* | | 0.000 | 0.000 | 0.000 | *0.07* | | 0.000 | 0.000 | 0.000 | *0.000* | |
| *Switching Urbanity* | 0.002 | 0.010 | 0.012 | *9.35* | | 0.000 | 0.003 | 0.003 | *0.47* | | 0.025 | 0.021 | 0.045 | *8.20* | | 0.003 | 0.002 | 0.004 | *16.41* | |
| *Switching Shore* | 0.000 | 0.001 | 0.001 | *0.73* | | 0.000 | 0.000 | 0.001 | *0.12* | | 0.002 | 0.001 | 0.003 | *0.53* | | 0.000 | 0.000 | 0.001 | *1.95* | |
| *Period* | -0.012 | 0.026 | 0.013 | *10.65* | | 0.004 | -0.001 | 0.003 | *0.51* | | 0.016 | 0.015 | 0.031 | *5.71* | | 0.005 | 0.004 | 0.008 | *32.03* | |
| *R² marginal* | 0.12 | | | | 0.57 | | | | | 0.55 | | | | | 0.03 | | | | |  |
| *R² conditional* | 0.35 | | | | 0.60 | | | | | 0.56 | | | | | 0.12 | | | | |  |

The table shows a matrix containing the unique (“Uniq.”), average shared (“Avg. share”), individual effect (“Indiv.”) and individual contribution percentage (“I. Perc”) for each predictor. The individual effect of a predictor is the sum of its unique effect and average shared effect. To represent the proportion of variance in the dependent variable that is explained by the independent variables, we also include the overall marginal R² (given by the fixed effects of the final model) and conditional R² (given by the fixed effects and random effects of the final model). The models were adapted for the “glmm.hp” package. For AFR, the partitioned values are shown for the model without the interaction between “Switching Urbanity” and “Switching Shore”. For NO, LRS and Fertile Years the R² values calculated by the trigamma method are shown. “—” means that the variable was not included in the full model.
