# Supplementary material for: Early and adult life environmental effects on reproductive performance in preindustrial women
Source: PLoS One. 2024 Oct 28;19(10):e0290212. doi: 10.1371/journal.pone.0290212 (PMC11515999; doi:10.1371/journal.pone.0290212)
Supplement: S6 Table — (DOCX) [file pone.0290212.s016.docx]

**S6 Table. Sequential models**

|  |  |
| --- | --- |
|  | Response variable ~ **Birth environment** + Wave front + (Fertile Years) + Distance + Period + (1 \| FamilyID) + (1 \| yearb) |
|  | Response variable ~ **Adult life environment** + Wave front + (Fertile Years) + Distance + Period + (1 \| FamilyID) + (1 \| yearb) |
|  | Response variable ~ **Switching Shore** + Wave front + (Fertile Years) + Distance + Period + (1 \| FamilyID) + (1 \| yearb) |
|  | Response variable ~ **Switching Urbanity** + Wave front + (Fertile Years) + Distance + Period + (1 \| FamilyID) + (1 \| yearb) |
|  | Response variable ~ **Birth environment + Adult life environment** + Wave front + (Fertile Years) + Distance + Period + (1 \| FamilyID) + (1 \| yearb) |
|  | Response variable ~ **Birth environment + Switching Shore** + Wave front + (Fertile Years) + Distance + Period + (1 \| FamilyID) + (1 \| yearb) |
|  | Response variable ~ **Birth environment + Switching Urbanity** + Wave front + (Fertile Years) + Distance + Period + (1 \| FamilyID) + (1 \| yearb) |
|  | Response variable ~ **Birth environment + Switching Urbanity** + **Switching Shore** + Wave front + (Fertile Years) + Distance + Period + (1 \| FamilyID) + (1 \| yearb) |
