# Supplementary material for: Early and adult life environmental effects on reproductive performance in preindustrial women
Source: PLoS One. 2024 Oct 28;19(10):e0290212. doi: 10.1371/journal.pone.0290212 (PMC11515999; doi:10.1371/journal.pone.0290212)
Supplement: S7 Table — (DOCX) [file pone.0290212.s017.docx]

|  | model | R2m | R2c |
| --- | --- | --- | --- |
| AFR | 1. Birth Environment | 0.122 | 0.348 |
|  | 1. Adult life environment | 0.071 | 0.300 |
|  | 1. Switching Shore | 0.068 | 0.295 |
|  | 1. Switching Urbanity | 0.108 | 0.331 |
|  | 1. Birth environment + Adult life environment | 0.123 | 0.349 |
|  | 1. Birth environment + Switching Shore | 0.122 | 0.348 |
|  | 1. Birth environment + Switching Urbanity | 0.124 | 0.350 |
|  | 1. Birth environment + Switching Urbanity*Switching Shore | **0.125** | **0.350** |
| NO | 1. Birth Environment | 0.583 | 0.611 |
|  | 1. Adult life environment | 0.584 | 0.612 |
|  | 1. Switching Shore | 0.584 | 0.612 |
|  | 1. Switching Urbanity | 0.584 | 0.611 |
|  | 1. Birth environment + Adult life environment | 0.027 | 0.492 |
|  | 1. Birth environment + Switching Shore | 0.584 | 0.611 |
|  | 1. Birth environment + Switching Urbanity | 0.584 | 0.611 |
|  | 1. Birth environment + Switching Urbanity*Switching Shore | **0.584** | **0.611** |
| LRS | 1. Birth Environment | 0.544 | 0.579 |
|  | 1. Adult life environment | 0.571 | 0.584 |
|  | 1. Switching Shore | 0.546 | 0.580 |
|  | 1. Switching Urbanity | 0.566 | 0.583 |
|  | 1. Birth environment + Adult life environment | 0.168 | 0.466 |
|  | 1. Birth environment + Switching Shore | 0.546 | 0.580 |
|  | 1. Birth environment + Switching Urbanity | 0.570 | 0.584 |
|  | 1. Birth environment + Switching Urbanity*Switching Shore | **0.572** | **0.585** |

**S7 Table. Marginal and conditional R^2^ of the sequential models, for Age at First Reproduction (AFR), Number of offspring (NO), and Lifetime reproductive success (LRS).**
