# Supplementary material for: Early and adult life environmental effects on reproductive performance in preindustrial women
Source: PLoS One. 2024 Oct 28;19(10):e0290212. doi: 10.1371/journal.pone.0290212 (PMC11515999; doi:10.1371/journal.pone.0290212)
Supplement: S8 Table — (DOCX) [file pone.0290212.s018.docx]

**S8 Table. Pairwise comparison between the different categories of Birth Environment for Age at First Reproduction (AFR) and Lifetime reproductive success (LRS).**

| Category 1 | Category 2 | AFR | | | LRS | | |
| --- | --- | --- | --- | --- | --- | --- | --- |
|  |  | Estimate | SE | P value | Estimate | SE | P value |
| Rural South | **Rural North** | 0.050 | 0.140 | 0.985 | 1.004 | 0.021 | 0.998 |
| Rural South | **Urban South** | 3.571 | 0.529 | **<0.001** | 1.377 | 0.104 | **<0.001** |
| Rural South | **Urban North** | 4.935 | 0.491 | **<0.001** | 1.353 | 0.101 | **<0.001** |
| Rural North | **Urban South** | 3.521 | 0.515 | **<0.001** | 1.372 | 0.100 | **<0.001** |
| Rural North | **Urban North** | 4.886 | 0.467 | **<0.001** | 1.348 | 0.095 | **<0.001** |
| Urban South | **Urban North** | 1.364 | 0.344 | **<0.001** | 0.983 | 0.041 | 0.976 |
