# Supplementary material for: Early and adult life environmental effects on reproductive performance in preindustrial women
Source: PLoS One. 2024 Oct 28;19(10):e0290212. doi: 10.1371/journal.pone.0290212 (PMC11515999; doi:10.1371/journal.pone.0290212)
Supplement: S9 Table — (DOCX) [file pone.0290212.s019.docx]

**S9 Table. Pairwise comparison between the different categories of the interaction between Switching Urbanity and Switching Shore for Age at First Reproduction (AFR).**

| Category 1 | Category 2 | AFR | | |
| --- | --- | --- | --- | --- |
|  |  | Estimate | SE | P value |
| Same Urbanity & Same Shore | **Urban to Rural & Same Shore** | -0.276 | 0.484 | *1.000* |
| Same Urbanity & Same Shore | **Rural to Urban & Same Shore** | -0.404 | 0.183 | *0.404* |
| Same Urbanity & Same Shore | **Same Urbanity & North to South** | -0.033 | 0.170 | *1.000* |
| Same Urbanity & Same Shore | **Urban to Rural and North to South** | -0.663 | 0.527 | *0.943* |
| Same Urbanity & Same Shore | **Rural to Urban and North to South** | -0.494 | 0.406 | *0.953* |
| Same Urbanity & Same Shore | **Same Urbanity & South to North** | 0.109 | 0.192 | *1.000* |
| Same Urbanity & Same Shore | **Urban to Rural and South to North** | -0.613 | 0.677 | *0.993* |
| Same Urbanity & Same Shore | **Rural to Urban and South to North** | -1.414 | 0.409 | ***0.016*** |
| Urban to Rural & Same Shore | **Rural to Urban & Same Shore** | -0.127 | 0.504 | *1.000* |
| Urban to Rural & Same Shore | **Same Urbanity & North to South** | 0.244 | 0.474 | *1.000* |
| Urban to Rural & Same Shore | **Urban to Rural and North to South** | -0.387 | 0.400 | *0.989* |
| Urban to Rural & Same Shore | **Rural to Urban and North to South** | -0.218 | 0.634 | *1.000* |
| Urban to Rural & Same Shore | **Same Urbanity & South to North** | 0.385 | 0.506 | *0.998* |
| Urban to Rural & Same Shore | **Urban to Rural and South to North** | -0.337 | 0.505 | *0.999* |
| Urban to Rural & Same Shore | **Rural to Urban and South to North** | -1.138 | 0.636 | *0.690* |
| Rural to Urban & Same Shore | **Same Urbanity & North to South** | 0.371 | 0.224 | *0.772* |
| Rural to Urban & Same Shore | **Urban to Rural and North to South** | -0.259 | 0.547 | *1.000* |
| Rural to Urban & Same Shore | **Rural to Urban and North to South** | -0.090 | 0.430 | *1.000* |
| Rural to Urban & Same Shore | **Same Urbanity & South to North** | 0.513 | 0.248 | *0.494* |
| Rural to Urban & Same Shore | **Urban to Rural and South to North** | -0.209 | 0.692 | *1.000* |
| Rural to Urban & Same Shore | **Rural to Urban and South to North** | -1.010 | 0.437 | *0.336* |
| Same Urbanity & North to South | **Urban to Rural and North to South** | -0.630 | 0.512 | *0.950* |
| Same Urbanity & North to South | **Rural to Urban and North to South** | -0.461 | 0.410 | *0.970* |
| Same Urbanity & North to South | **Same Urbanity & South to North** | 0.142 | 0.249 | *1.000* |
| Same Urbanity & North to South | **Urban to Rural and South to North** | -0.580 | 0.671 | *0.995* |
| Same Urbanity & North to South | **Rural to Urban and South to North** | -1.381 | 0.438 | ***0.043*** |
| Urban to Rural and North to South | **Rural to Urban and North to South** | 0.169 | 0.662 | *1.000* |
| Urban to Rural and North to South | **Same Urbanity & South to North** | 0.772 | 0.550 | *0.897* |
| Urban to Rural and North to South | **Urban to Rural and South to North** | 0.050 | 0.637 | *1.000* |
| Urban to Rural and North to South | **Rural to Urban and South to North** | -0.751 | 0.668 | *0.971* |
| Rural to Urban and North to South | **Same Urbanity & South to North** | 0.603 | 0.445 | *0.914* |
| Rural to Urban and North to South | **Urban to Rural and South to North** | -0.119 | 0.790 | *1.000* |
| Rural to Urban and North to South | **Rural to Urban and South to North** | -0.920 | 0.568 | *0.795* |
| Same Urbanity & South to North | **Urban to Rural and South to North** | -0.722 | 0.690 | *0.981* |
| Same Urbanity & South to North | **Rural to Urban and South to North** | -1.523 | 0.431 | ***0.012*** |
| Urban to Rural and South to North | **Rural to Urban and South to North** | -0.801 | 0.792 | *0.985* |
