# Supplementary material for: Early and adult life environmental effects on reproductive performance in preindustrial women
Source: PLoS One. 2024 Oct 28;19(10):e0290212. doi: 10.1371/journal.pone.0290212 (PMC11515999; doi:10.1371/journal.pone.0290212)
Supplement: S10 Table — (DOCX) [file pone.0290212.s020.docx]

| Category 1 | Category 2 | LRS | | |
| --- | --- | --- | --- | --- |
|  |  | Estimate | SE | P value |
| Same Urbanity | **Urban to Rural** | 0.735 | 0.048 | ***<0.001*** |
| Same Urbanity | **Rural to Urban** | 1.409 | 0.033 | ***<0.001*** |
| Urban to Rural | **Rural to Urban** | 1.916 | 0.134 | ***<0.001*** |

**S10 Table. Pairwise comparison between the different categories of Switching Urbanity for Lifetime reproductive success (LRS).**
