# Supplementary material for: Early and adult life environmental effects on reproductive performance in preindustrial women
Source: PLoS One. 2024 Oct 28;19(10):e0290212. doi: 10.1371/journal.pone.0290212 (PMC11515999; doi:10.1371/journal.pone.0290212)
Supplement: S11 Table — (DOCX) [file pone.0290212.s021.docx]

**S11 Table. Pairwise comparison between the different categories of Switching Shore for Lifetime reproductive success (LRS).**

| Category 1 | Category 2 | LRS | | |
| --- | --- | --- | --- | --- |
|  |  | Estimate | SE | P value |
| Same Shore | **North to South** | 0.982 | 0.020 | *0.663* |
| Same Shore | **South to North** | 1.089 | 0.028 | ***0.003*** |
| North to South | **South to North** | 1.108 | 0.036 | ***0.005*** |
