# Supplementary material for: Early and adult life environmental effects on reproductive performance in preindustrial women
Source: PLoS One. 2024 Oct 28;19(10):e0290212. doi: 10.1371/journal.pone.0290212 (PMC11515999; doi:10.1371/journal.pone.0290212)
Supplement: S1 Data — (HTML) [file pone.0290212.s022.html]

Models.Reviewed


# Models.Reviewed

#### 2024-06-17

Output shown in Table 2 and Table S4

Libraries

```
library(glmmTMB)
library(readr)
library(ggplot2)
library(ggeffects)
library(cowplot)
library(ggplot2)
library(ggpubr)
library(dplyr)
library(DHARMa)
library(MuMIn)
library(glmm.hp)
```

The following files are needed

- Subset.1750
- Subset.1729

The variables in the models are:

-Fixed effects:

–urb\_riv\_parishb: Environment of Birth

–switch\_urbain\_adult: Direction of the change of urbanity between the
urbanity of the parish of birth and the urbanity with more reproductive
events.

–switch\_rive\_adult: Direction of the change of river shore between
the shore of the parish of birth and the shore with more reproductive
events.

–dist.km\_FR: Distance in kilometers between the parish of birth and
the parish of first reproduction

–wavefront: Time in years between the foundation of the parish of
birth and the date of first reproduction

–fertile.y: Number of fertile years, which are the years during which
both the individual (woman) and her husband were alive, between the date
of the wedding and the 45th birthday of the individual

–Period.hogei: The variable Period is categorical and describes five
intervals, given the date of birth of the woman. Each interval spans 20
years, except the first one which spans 30 years: 1640-1669, 1670-1689,
1690-1709, 1710-1729 and after 1730. The fifth period does not appear in
the analysis for LRS, since this subset only includes women born before
1729.

-Random effects:

–year.b: Year of birth of the individual

–FamilyID: Family Identity, union of the parents, shared by the
siblings with the same mother and father (result of the same
marriage)

# 1) Main Analysis

## AFR

AFR is the Age at First Reproduction: age at with the woman’s first
child was born

### Full model

```
mr_AFR_1 <- glmmTMB(AFR~  +urb_riv_parishb  +  scale(wavefront) + scale(dist.km_FR) + switch_urbain_adult + switch_rive_adult + switch_urbain_adult* switch_rive_adult  + Period.hogei + (1 | FamilyID) + (1 | yearb) , family = "gaussian",data = Subset.1750)

summary(mr_AFR_1)

car::Anova(mr_AFR_1, type = 3, test = "Chisq")

r2.mr_AFR_1 <-r.squaredGLMM(mr_AFR_1)
```

### Model without interaction

```
mr_AFR_2 <- glmmTMB(AFR~  +urb_riv_parishb  +  scale(wavefront) + scale(dist.km_FR) + switch_urbain_adult + switch_rive_adult   + Period.hogei + (1 | FamilyID) + (1 | yearb) , family = "gaussian",data = Subset.1750)

summary(mr_AFR_2)

car::Anova(mr_AFR_2, type = 3, test = "Chisq")
```

## NO

NO is the Number of Offspring: number of offspring born to each
woman

### Full model

```
mr_NO_1 <- glmmTMB(NO~  urb_riv_parishb  +  scale(wavefront) + scale(fertile.y) + scale(dist.km_FR) + switch_urbain_adult+ switch_rive_adult + switch_urbain_adult* switch_rive_adult + Period.hogei + (1 | FamilyID) + (1 | yearb) , family = "poisson",data = Subset.1750)
summary(mr_NO_1)

car::Anova(mr_NO_1, type = 3, test = "Chisq")

r2.mr_NO_1 <-r.squaredGLMM(mr_NO_1)
```

### Model without interaction

```
mr_NO_2 <- glmmTMB(NO~  urb_riv_parishb  +  scale(wavefront) + scale(fertile.y) + scale(dist.km_FR) + switch_urbain_adult+ switch_rive_adult +  Period.hogei + (1 | FamilyID) + (1 | yearb) , family = "poisson",data = Subset.1750)

summary(mr_NO_2)


car::Anova(mr_NO_2, type = 3, test = "Chisq")
```

## LRS before 1729

LRS is Lifetime Reproductive Success: number of offspring who
survived to adulthood (15yo). A different subset is used for this
analysis (until 1729 instead of 1750) to ensure that the survival of the
offspring is accurately registered.

### Full model

```
mr_LRS_1 <- glmmTMB(LRS.alt~  urb_riv_parishb  +  scale(wavefront) + scale(fertile.y) + scale(dist.km_FR) + switch_urbain_adult+ switch_rive_adult + switch_urbain_adult* switch_rive_adult + Period.hogei + (1 | FamilyID) + (1 | yearb) , family = "poisson",data = Subset.1729)

summary(mr_LRS_1)

car::Anova(mr_LRS_1, type = 3, test = "Chisq")


r2.mr_LRS_1 <-r.squaredGLMM(mr_LRS_1)
```

### Model without interaction

```
mr_LRS_2 <- glmmTMB(LRS.alt~  urb_riv_parishb  +  scale(wavefront) + scale(fertile.y) + scale(dist.km_FR) + switch_urbain_adult+ switch_rive_adult + Period.hogei + (1 | FamilyID) + (1 | yearb) , family = "poisson",data = Subset.1729)

summary(mr_LRS_2)


car::Anova(mr_LRS_2, type = 3, test = "Chisq")
```

# 2) Additional Analysis

## Fertile years

Fertile years is the number of years during which both the woman and
her husband were alive between the date of marriage and the 45th
birthday of the woman

### Full model

```
mr_FY_1 <- glmmTMB(fertile.y ~  urb_riv_parishb  +  scale(wavefront) + scale(dist.km_FR) + switch_urbain_adult+ switch_rive_adult + switch_urbain_adult* switch_rive_adult + Period.hogei +(1 | FamilyID) + (1 | yearb) , family = "gaussian",data = Subset.1750)

summary(mr_FY_1)

car::Anova(mr_FY_1, type = 3, test = "Chisq")

r2.mr_FY_1 <-r.squaredGLMM(mr_FY_1)
```

### Model without interaction

```
mr_FY_2 <- glmmTMB(fertile.y ~  urb_riv_parishb  +  scale(wavefront) + scale(dist.km_FR) + switch_urbain_adult+ switch_rive_adult +  Period.hogei +(1 | FamilyID) + (1 | yearb) , family = "gaussian",data = Subset.1750)

summary(mr_FY_2)


car::Anova(mr_FY_2, type = 3, test = "Chisq")
```

## Proportion between NO and LRS

The proportion between LRS and NO (LRS/NO) indicates the proportion
of offspring who survived to adulthood

### Full model

```
mr_cb_1 <- glmmTMB(cbind(LRS.alt,LRF.alt)~  urb_riv_parishb  +  scale(wavefront) + scale(fertile.y) + scale(dist.km_FR) + switch_urbain_adult+ switch_rive_adult + switch_urbain_adult* switch_rive_adult + Period.hogei + (1 | FamilyID) + (1 | yearb) , family = betabinomial(link = "logit"),data = Subset.1750)

summary(mr_cb_1)

car::Anova(mr_cb_1, type = 3, test = "Chisq")

r2.mr_cb_1 <-r.squaredGLMM(mr_cb_1)
```

### Model without interaction

```
mr_cb_2 <- glmmTMB(cbind(LRS.alt,LRF.alt)~  urb_riv_parishb  +  scale(wavefront) + scale(fertile.y) + scale(dist.km_FR) + switch_urbain_adult+ switch_rive_adult  + Period.hogei + (1 | FamilyID) + (1 | yearb) , family = betabinomial(link = "logit"),data = Subset.1750)

summary(mr_cb_2)

car::Anova(mr_cb_2, type = 3, test = "Chisq")
```

# 3) Sensitivity Analysis

## Agem

Agem is the age at marriage: age of the woman when the wedding with
her husband was celebrated, as an indicator of the onset of reproductive
potential.

### Full model

```
mr_agem_1 <- glmmTMB(agem~  +urb_riv_parishb  +  scale(wavefront) + scale(dist.km_FR) + switch_urbain_adult+ switch_rive_adult + switch_urbain_adult* switch_rive_adult+ Period.hogei + (1 | FamilyID) + (1 | yearb) , family = "gaussian",data = Subset.1750)

summary(mr_agem_1)

car::Anova(mr_agem_1, type = 3, test = "Chisq")

r2.mr_agem_1 <-r.squaredGLMM(mr_agem_1)
```

### Model without interaction

```
mr_agem_2 <- glmmTMB(agem~  +urb_riv_parishb  +  scale(wavefront) + scale(dist.km_FR) + switch_urbain_adult+ switch_rive_adult + Period.hogei + (1 | FamilyID) + (1 | yearb) , family = "gaussian",data = Subset.1750)

summary(mr_agem_2)


car::Anova(mr_agem_2, type = 3, test = "Chisq")
```

## AFR with only woman older than 15

### Creating subset

```
Subset.1750_plus15 <- Subset.1750[Subset.1750$AFR > 15, ]
```

### Full model

```
mr_AFR_plus15_1 <- glmmTMB(AFR~  +urb_riv_parishb  +  scale(wavefront) + scale(dist.km_FR) + switch_urbain_adult + switch_rive_adult + switch_urbain_adult* switch_rive_adult  + Period.hogei + (1 | FamilyID) + (1 | yearb) , family = "gaussian",data = Subset.1750_plus15)

summary(mr_AFR_plus15_1)

car::Anova(mr_AFR_plus15_1, type = 3, test = "Chisq")


r2.mr_AFR_plus15_1 <-r.squaredGLMM(mr_AFR_plus15_1)
```

### Model without interaction

```
mr_AFR_plus15_2 <- glmmTMB(AFR~  +urb_riv_parishb  +  scale(wavefront) + scale(dist.km_FR) + switch_urbain_adult + switch_rive_adult   + Period.hogei + (1 | FamilyID) + (1 | yearb) , family = "gaussian",data = Subset.1750_plus15)

summary(mr_AFR_plus15_2)

car::Anova(mr_AFR_plus15_2, type = 3, test = "Chisq")
```
