# Supplementary material for: Early and adult life environmental effects on reproductive performance in preindustrial women
Source: PLoS One. 2024 Oct 28;19(10):e0290212. doi: 10.1371/journal.pone.0290212 (PMC11515999; doi:10.1371/journal.pone.0290212)
Supplement: S2 Data — (HTML) [file pone.0290212.s023.html]

Partitioned.R


# Partitioned.R

#### 2024-06-19

Output shown in S5 Table

Libraries

```
library(glmmTMB)
library(readr)
library(ggplot2)
library(ggeffects)
library(cowplot)
library(ggplot2)
library(ggpubr)
library(dplyr)
library(DHARMa)
library(MuMIn)
library(glmm.hp)
```

The following files are needed

- Subset.1750
- Subset.1729

# 1) Main analyses

## AFR

```
#Model for glmm.hp

m_AFR_hp <- glmmTMB(AFR ~  urb_riv_parishb  +  wavefront + dist.km_FR + switch_urbain_adult+ switch_rive_adult + Period.hogei + (1 | FamilyID) + (1 | yearb) , data = Subset.1750)

summary(m_AFR_hp)

#Partitioned R^2

AFR_hp_result <-glmm.hp(m_AFR_hp)
```

## NO

```
#Model for glmm.hp

mr_NO_hp <- glmmTMB(NO~  urb_riv_parishb  +  wavefront + dist.km_FR + switch_urbain_adult+ switch_rive_adult + Period.hogei + fertile.y + (1 | FamilyID) + (1 | yearb) , family = "poisson",data = Subset.1750)


#Partitioned R^2
mr_NO_hp_result <-glmm.hp(mr_NO_hp )

mr_NO_hp_result
```

## LRS before 1729

```
#Model for glmm.hp

mr_LRS_hp <- glmmTMB(LRS.alt~  urb_riv_parishb  +  wavefront + dist.km_FR + switch_urbain_adult+ switch_rive_adult + Period.hogei + fertile.y + (1 | FamilyID) + (1 | yearb) , family = "poisson",data = Subset.1729)


#Partitioned R^2
m_LRS.1729_hp_result <-glmm.hp(mr_LRS_hp)

m_LRS.1729_hp_result
```

# 2) Additional analysis

## Fertile years

```
#Model for glmm.hp

mr_FY_hp <- glmmTMB(fertile.y ~  urb_riv_parishb  +  wavefront + dist.km_FR + switch_urbain_adult+ switch_rive_adult + Period.hogei + (1 | FamilyID) + (1 | yearb) , family = "gaussian",data = Subset.1750)

#Partitioned R^2
m_FY_hp_result <-glmm.hp(mr_FY_hp)
```

# 3) Sensitivity analysis

## Age at Marriage

```
#Model for glmm.hp
mr_agem_hp <- glmmTMB(agem~  urb_riv_parishb  +  wavefront + dist.km_FR + switch_urbain_adult+ switch_rive_adult + Period.hogei + (1 | FamilyID) + (1 | yearb) , family = "gaussian",data = Subset.1750)

summary(mr_agem_hp)

#Partitioned R^2
agem_hp_result <-glmm.hp(mr_agem_hp)
```
