# Supplementary material for: Early and adult life environmental effects on reproductive performance in preindustrial women
Source: PLoS One. 2024 Oct 28;19(10):e0290212. doi: 10.1371/journal.pone.0290212 (PMC11515999; doi:10.1371/journal.pone.0290212)
Supplement: S3 Data — (HTML) [file pone.0290212.s024.html]

Plots.review


# Plots.review

#### 2024-06-17

Output shown in the figures in the manuscript and the supplementary
material.

Libraries

```
library(dplyr)
library(glmmTMB)
library(readr)
library(ggplot2)
library(ggeffects)
library(cowplot)
library(ggplot2)
library(ggpubr)
library(stringr)
library(emmeans)
library(multcomp)
library(ggh4x)
library(gridExtra)
library(patchwork)
library(MuMIn)
library(glmm.hp)
library(tidyr)
```

The following files are needed

- Subset.1750
- Subset.1729

# 1) Main Analysis

## AFR

### Model

```
mr_AFR_1 <- glmmTMB(AFR~  +urb_riv_parishb  +  scale(wavefront) + scale(dist.km_FR) + switch_urbain_adult + switch_rive_adult + switch_urbain_adult* switch_rive_adult  + Period.hogei + (1 | FamilyID) + (1 | yearb) , family = "gaussian",data = Subset.1750)

summary(mr_AFR_1)


car::Anova(mr_AFR_1, type = 3, test = "Chisq")

mr_AFR_2 <- glmmTMB(AFR~  +urb_riv_parishb  +  scale(wavefront) + scale(dist.km_FR) + switch_urbain_adult + switch_rive_adult   + Period.hogei + (1 | FamilyID) + (1 | yearb) , family = "gaussian",data = Subset.1750)

summary(mr_AFR_2)


car::Anova(mr_AFR_2, type = 3, test = "Chisq")
```

### Tukey test

```
#AFR


e.afr_pb <-emmeans(mr_AFR_1  , list(pairwise ~ urb_riv_parishb), adjust = "tukey")
cld.afr_pb<-cld(e.afr_pb , Letter="abcdefg")

e.afr_int <-emmeans(mr_AFR_1  , list(pairwise ~ switch_urbain_adult* switch_rive_adult), adjust = "tukey")
cld.afr_int<-cld(e.afr_int , Letter="abcdefg")

e.afr_surb <-emmeans(mr_AFR_2  , list(pairwise ~ switch_urbain_adult), adjust = "tukey")
cld.afr_surb<-cld(e.afr_surb , Letter="abcdefg")

e.afr_sriv <-emmeans(mr_AFR_2  , list(pairwise ~ switch_rive_adult), adjust = "tukey")
cld.afr_sriv<-cld(e.afr_sriv , Letter="abcdefg")
```

### Birth

```
p_AFR_pb <- ggpredict(mr_AFR_1 , terms = c("urb_riv_parishb"))

p_AFR_pb$var <- "Environment of birth"

p_AFR_pb[c('Urbanity', 'Shore')] <- str_split_fixed(p_AFR_pb$x, '_', 2)

p_tuk_AFR_pb <- merge(p_AFR_pb ,cld.afr_pb, by.x= "x" , by.y= "urb_riv_parishb")

pl.AFR.pb <- ggplot(p_tuk_AFR_pb,aes(y=predicted,x=Urbanity,ymin=conf.low,ymax=conf.high,color=Shore))+   geom_pointrange(position = position_dodge(0.3),size=0.8) +
       scale_color_manual("Shore of birth",labels = c("South (Good)", "North (Bad)"), values = c("1.South" = "blue", "2.North" = "red")) + #to add the colors and the legend of the colors
  theme(plot.title = element_text(size=20,hjust = 0.5,face = "bold"),axis.title.y = element_text(size=22),axis.text.y = element_text(size=15),axis.title.x  = element_text(size=22),axis.text.x  = element_text(size=17),legend.title = element_text(size=22),legend.text =  element_text(size=20),panel.background = element_rect(fill = "white", colour = "grey50"),  panel.grid.major = element_line(colour = "grey90"),plot.tag=element_text(size=20))  +
  xlab("") +
  ylab("Age at First Reproduction") +
 scale_y_continuous(limits=c(15.5,26),breaks = seq(16,26,by = 1)) + 
  scale_x_discrete(limits=c("1.Rural","2.Urban"),labels =  c("Rural\n(Good)", "Urban\n(Bad)"))+
  geom_text(aes(label = .group ), position = position_dodge(width = 0.35),vjust = -1.8, hjust = 1.0, size = 5 )+
  labs(tag = "A") 

plot(pl.AFR.pb)
```

### Interaction Switching (Fig.2)

```
#prediction of the two variables for switching (Swithing urbanity and switching shore)

p_AFR_int<- ggpredict(mr_AFR_1 , terms = c("switch_urbain_adult","switch_rive_adult"))


#name changing necessary to do the beautiful plot
colnames(p_AFR_int)[colnames(p_AFR_int) == "x"]  <- "switch_urbain_adult"
colnames(p_AFR_int)[colnames(p_AFR_int) == "group"] <- "switch_rive_adult"

p_tuk_AFR_int <- merge(p_AFR_int ,cld.afr_int, by=c("switch_urbain_adult", "switch_rive_adult"))

#beautiful grey, orange and green plot
pl.AFR.int <- ggplot(p_tuk_AFR_int,aes(y=predicted,x=switch_urbain_adult,ymin=conf.low,ymax=conf.high,colour=switch_rive_adult,fill=switch_rive_adult,shape=switch_urbain_adult))+   geom_pointrange(position = position_dodge(0.3),size=0.8)+ scale_shape_manual("Switching Urbanity",labels = c("Same urbanity (Same conditions)", "Urban to Rural (Bad to Good)","Rural to Urban (Good to Bad)"), values = c("1.same" = 21, "2.U-R" = 24,"3.R-U" = 25)) +
  scale_color_manual("Switching Shore",labels = c("Same shore (Same conditions)", "North to South (Bad to Good)","South to North (Good to Bad)"), values = c("1.same" = "grey", "2.N-S" = "green","3.S-N" = "orange")) +
  scale_fill_manual("Switching Shore",labels = c("Same shore (Same conditions)", "North to South (Bad to Good)","South to North (Good to Bad)"), values = c("1.same" = "grey", "2.N-S" = "green","3.S-N" = "orange")) + #scale_shape_manual(name  = "Switching Urbanity", breaks=c("1.same", "2.U-R","3.R-U"),labels=c("1.same"="Same urbanity (Same conditions)", "2.U-R"="Urban to Rural (Bad to Good)","3.R-U"="Rural to Urban (Good to Bad)"),values=c("1.same"=21,"2.U-R"=24,"3.R-U"=25))+
  
  theme(plot.title = element_text(size=20,hjust = 0.5,face = "bold"),axis.title.y = element_text(size=22),axis.text.y = element_text(size=12),axis.title.x  = element_text(size=20),axis.text.x  = element_text(size=14),legend.title = element_text(size=16),legend.text =  element_text(size=14),panel.background = element_rect(fill = "white", colour = "grey50"),  panel.grid.major = element_line(colour = "grey90"),plot.tag=element_text(size=20)) +
  xlab("Switching Urbanity") + ylab("Age at First Reproduction") +
 scale_y_continuous(limits=c(15.5,26),breaks = seq(16,26,by = 1)) + 
  scale_x_discrete(limits=c("1.same","2.U-R","3.R-U"),labels =  c("Same Urbanity\n(Same conditions)", "Urban to Rural\n(Bad to Good)", "Rural to Urban\n(Good to Bad)"))+
  geom_text(aes(label = .group ), position = position_dodge(width = 0.35),vjust = -0.8, hjust = 0.5, size = 5 ) 

plot(pl.AFR.int)
```

```
ggsave("Figure2.tiff",width=10, height=6, dpi=600)
```

### Distance

```
p_AFR_dist <- ggpredict(mr_AFR_1 , terms = "dist.km_FR [all]")
pl.AFR.dist <-ggplot(p_AFR_dist, aes(x, predicted)) +
  geom_line(colour="blue") +
  geom_ribbon(aes(ymin = conf.low, ymax = conf.high), alpha = .1)+
  theme(plot.title = element_text(size=20,hjust = 0.5,face = "bold"),axis.title.y = element_text(size=22),axis.text.y = element_text(size=12),axis.title.x  = element_text(size=20),axis.text.x  = element_text(size=17),legend.title = element_text(size=22),legend.text =  element_text(size=20),panel.background = element_rect(fill = "white", colour = "grey50"),  panel.grid.major = element_line(colour = "grey90"),plot.tag=element_text(size=20)) +    scale_y_continuous(limits=c(15.5,26),breaks = seq(16,26,by = 1)) +  
   xlab("Distance (Km)") + ylab("Age at First Reproduction")+
  labs(tag = "A")

plot(pl.AFR.dist)
```

## NO

### Model

```
mr_NO_1 <- glmmTMB(NO~  urb_riv_parishb  +  scale(wavefront) + scale(fertile.y) + scale(dist.km_FR) + switch_urbain_adult+ switch_rive_adult + switch_urbain_adult* switch_rive_adult + Period.hogei + (1 | FamilyID) + (1 | yearb) , family = "poisson",data = Subset.1750)

summary(mr_NO_1)


car::Anova(mr_NO_1, type = 3, test = "Chisq")


mr_NO_2 <- glmmTMB(NO~  urb_riv_parishb  +  scale(wavefront) + scale(fertile.y) + scale(dist.km_FR) + switch_urbain_adult+ switch_rive_adult +  Period.hogei + (1 | FamilyID) + (1 | yearb) , family = "poisson",data = Subset.1750)

summary(mr_NO_2)


car::Anova(mr_NO_2, type = 3, test = "Chisq")
```

### Tukey test

```
#NO

e.NO_pb <-emmeans(
mr_NO_1 , list(pairwise ~ urb_riv_parishb), adjust = "tukey")
cld.NO_pb<-cld(e.NO_pb , Letter="abcdefg")

e.NO_int <-emmeans(mr_NO_1   , list(pairwise ~ switch_urbain_adult* switch_rive_adult), adjust = "tukey")
cld.NO_int<-cld(e.NO_int , Letter="abcdefg")

e.NO_sriv <-emmeans(
mr_NO_2 , list(pairwise ~ switch_rive_adult), adjust = "tukey")
cld.NO_sriv<-cld(e.NO_sriv , Letter="abcdefg")


e.NO_surb <-emmeans(
mr_NO_2 , list(pairwise ~ switch_urbain_adult), adjust = "tukey")
cld.NO_surb<-cld(e.NO_surb , Letter="abcdefg")
```

### Birth

```
p_NO_pb <- ggpredict(mr_NO_1, terms = c("urb_riv_parishb"))

p_NO_pb$var <- "Environment of birth"

p_NO_pb[c('Urbanity', 'Shore')] <- str_split_fixed(p_NO_pb$x, '_', 2)

p_tuk_NO_pb <- merge(p_NO_pb ,cld.NO_pb, by.x= "x" , by.y= "urb_riv_parishb")

pl.NO.pb <- ggplot(p_tuk_NO_pb,aes(y=predicted,x=Urbanity,ymin=conf.low,ymax=conf.high,color=Shore))+   geom_pointrange(position = position_dodge(0.3),size=0.8) +
       scale_color_manual("Shore of birth",labels = c("South (Good)", "North (Bad)"), values = c("1.South" = "blue", "2.North" = "red")) + #to add the colors and the legend of the colors
  theme(plot.title = element_text(size=20,hjust = 0.5,face = "bold"),axis.title.y = element_text(size=22),axis.text.y = element_text(size=15),axis.title.x  = element_text(size=22),axis.text.x  = element_text(size=17),legend.title = element_text(size=22),legend.text =  element_text(size=20),panel.background = element_rect(fill = "white", colour = "grey50"),  panel.grid.major = element_line(colour = "grey90"),plot.tag=element_text(size=20))  +
  xlab("Urbanity of birth") +
  ylab("Number of Offspring") +
   scale_y_continuous(limits=c(2,8),breaks = seq(2,8,by = 1)) +   
  scale_x_discrete(limits=c("1.Rural","2.Urban"),labels =  c("Rural\n(Good)", "Urban\n(Bad)"))+
  geom_text(aes(label = .group ), position = position_dodge(width = 0.35),vjust = -1.3, hjust = 0.9, size = 5 ) +
  labs(tag = "A")

plot(pl.NO.pb)
```

### Swithing Urbanity

```
p_NO_surb<- ggpredict(mr_NO_2, terms = c("switch_urbain_adult"))


#name changing necessary to do the beautiful plot
colnames(p_NO_surb)[colnames(p_NO_surb) == "x"] <- "switch_urbain_adult"

p_tuk_NO_surb <- merge(p_NO_surb ,cld.NO_surb, by= "switch_urbain_adult")


pl.NO.surb <- ggplot(p_tuk_NO_surb ,aes(y=predicted,x=switch_urbain_adult,ymin=conf.low,ymax=conf.high,shape=switch_urbain_adult))+   geom_pointrange(position = position_dodge(0.3),size=0.8)+
  theme(
    plot.title = element_text(size = 20, hjust = 0.5, face = "bold"),
    axis.title.y = element_text(size = 22),
    axis.text.y = element_text(size = 15),
    axis.title.x = element_text(size = 20),
    axis.text.x = element_text(size = 17),
    legend.position = "none",  # Remove the legend
    panel.background = element_rect(fill = "white", colour = "grey50"),
    panel.grid.major = element_line(colour = "grey90"),
    plot.tag = element_text(size = 20))+
  
  xlab("Switching Urbanity") + ylab("Number of Offspring") +
  scale_y_continuous(limits=c(2,8),breaks = seq(2,8,by = 1)) +     
  scale_x_discrete(limits=c("1.same","2.U-R","3.R-U"),labels =  c("Same Urbanity\n(Same conditions)", "Urban to Rural \n(Bad to Good)", "Rural to Urban\n(Good to Bad)"))+
  geom_text(aes(label = .group ), position = position_dodge(width = 0.35),vjust = -1, hjust = 1.5, size = 5 )+ scale_shape_manual("Switching Urbanity",labels = c("Same urbanity (Same conditions)", "Urban to Rural (Bad to Good)","Rural to Urban (Good to Bad)"), values = c("1.same" = 21, "2.U-R" = 24,"3.R-U" = 25)) + 
  labs(tag = "A")

plot(pl.NO.surb)
```

### Swithing Shore

```
p_NO_sriv<- ggpredict(mr_NO_2, terms = c("switch_rive_adult"))


#name changing necessary to do the beautiful plot
colnames(p_NO_sriv)[colnames(p_NO_sriv) == "x"] <- "switch_rive_adult"

p_tuk_NO_sriv <- merge(p_NO_sriv ,cld.NO_sriv, by= "switch_rive_adult")
pl.NO.sriv <- ggplot(p_tuk_NO_sriv, aes(y = predicted, x = switch_rive_adult, ymin = conf.low, ymax = conf.high, colour = switch_rive_adult)) +
  geom_pointrange(position = position_dodge(0.3), size = 0.8) +
  theme(
    plot.title = element_text(size = 20, hjust = 0.5, face = "bold"),
    axis.title.y = element_text(size = 22),
    axis.text.y = element_text(size = 15),
    axis.title.x = element_text(size = 20),
    axis.text.x = element_text(size = 17),
    legend.position = "none",  # Remove the legend
    panel.background = element_rect(fill = "white", colour = "grey50"),
    panel.grid.major = element_line(colour = "grey90"),
    plot.tag = element_text(size = 20))+
  xlab("Switching shore") + ylab("Number of Offspring") +
  scale_y_continuous(limits=c(2,8),breaks = seq(2,8,by = 1)) +   
  scale_x_discrete(
    limits = c("1.same", "2.N-S", "3.S-N"),
    labels = c("Same shore\n(Same conditions)", "North to South\n(Bad to Good)", "South to North\n(Good to Bad)")
  ) +
  geom_text(aes(label = .group), position = position_dodge(width = 0.35), vjust = -1, hjust = 1.5, size = 5) +
  scale_color_manual(
    "Switching Shore",
    labels = c("Same shore (Same conditions)", "North to South (Bad to Good)", "South to North (Good to Bad)"),
    values = c("1.same" = "grey", "2.N-S" = "green", "3.S-N" = "orange")
  ) +
  scale_fill_manual(
    "Switching Shore",
    labels = c("Same shore (Same conditions)", "North to South (Bad to Good)", "South to North (Good to Bad)"),
    values = c("1.same" = "grey", "2.N-S" = "green", "3.S-N" = "orange")
  ) +
  labs(tag = "B")


plot(pl.NO.sriv)
```

## LRS before 1729

LRS is the number of offspring who survived to adulthood

### Model

```
mr_LRS_1 <- glmmTMB(LRS.alt~  urb_riv_parishb  +  scale(wavefront) + scale(fertile.y) + scale(dist.km_FR) + switch_urbain_adult+ switch_rive_adult + switch_urbain_adult* switch_rive_adult + Period.hogei + (1 | FamilyID) + (1 | yearb) , family = "poisson",data = Subset.1729)

summary(mr_LRS_1)


car::Anova(mr_LRS_1, type = 3, test = "Chisq")

mr_LRS_2 <- glmmTMB(LRS.alt~  urb_riv_parishb  +  scale(wavefront) + scale(fertile.y) + scale(dist.km_FR) + switch_urbain_adult+ switch_rive_adult + Period.hogei + (1 | FamilyID) + (1 | yearb) , family = "poisson",data = Subset.1729)

summary(mr_LRS_2)


car::Anova(mr_LRS_2, type = 3, test = "Chisq")
```

### Tukey test

```
#LRS


e.LRS_pb <-emmeans(mr_LRS_1 , list(pairwise ~ urb_riv_parishb), adjust = "tukey", type = "response")
cld.LRS_pb<-cld(e.LRS_pb , Letter="abcdefg", type = "response")


e.LRS_pb2 <-emmeans(mr_LRS_2 , list(pairwise ~ urb_riv_parishb), adjust = "tukey", type = "response")
cld.LRS_pb2<-cld(e.LRS_pb2 , Letter="abcdefg", type = "response")


e.LRS_surb <-emmeans(mr_LRS_2 , list(pairwise ~ switch_urbain_adult), adjust = "tukey", type = "response")
cld.LRS_surb<-cld(e.LRS_surb , Letter="abcdefg", type = "response")

e.LRS_sriv <-emmeans(mr_LRS_2 , list(pairwise ~ switch_rive_adult), adjust = "tukey", type = "response")
cld.LRS_sriv<-cld(e.LRS_sriv , Letter="abcdefg", type = "response")
```

### Birth

```
p_LRS_pb <- ggpredict(mr_LRS_1, terms = c("urb_riv_parishb"))

p_LRS_pb$var <- "Environment of birth"

p_LRS_pb[c('Urbanity', 'Shore')] <- str_split_fixed(p_LRS_pb$x, '_', 2)

p_tuk_LRS_pb <- merge(p_LRS_pb ,cld.LRS_pb, by.x= "x" , by.y= "urb_riv_parishb")

pl.LRS.pb <- ggplot(p_tuk_LRS_pb,aes(y=predicted,x=Urbanity,ymin=conf.low,ymax=conf.high,color=Shore))+   geom_pointrange(position = position_dodge(0.3),size=0.8) +
       scale_color_manual("Shore of birth",labels = c("South (Good)", "North (Bad)"), values = c("1.South" = "blue", "2.North" = "red")) + #to add the colors and the legend of the colors
  theme(plot.title = element_text(size=20,hjust = 0.5,face = "bold"),axis.title.y = element_text(size=22),axis.text.y = element_text(size=15),axis.title.x  = element_text(size=22),axis.text.x  = element_text(size=17),legend.title = element_text(size=22),legend.text =  element_text(size=20),panel.background = element_rect(fill = "white", colour = "grey50"),  panel.grid.major = element_line(colour = "grey90"),plot.tag=element_text(size=20))  +
  xlab("") +
  ylab("Lifetime Reproductive Success") +
  scale_y_continuous(limits=c(2,8),breaks = seq(2,8,by = 1)) +    
  scale_x_discrete(limits=c("1.Rural","2.Urban"),labels =  c("Rural\n(Good)", "Urban\n(Bad)"))+
  geom_text(aes(label = .group ), position = position_dodge(width = 0.35),vjust = -1, hjust = 1.2, size = 5 ) +
  labs(tag = "B")

plot(pl.LRS.pb)
```

### Swithing Urbanity

```
p_LRS_surb<- ggpredict(mr_LRS_2, terms = c("switch_urbain_adult"))


#name changing necessary to do the beautiful plot
colnames(p_LRS_surb)[colnames(p_LRS_surb) == "x"] <- "switch_urbain_adult"

p_tuk_LRS_surb <- merge(p_LRS_surb ,cld.LRS_surb, by= "switch_urbain_adult")


pl.LRS.surb <- ggplot(p_tuk_LRS_surb ,aes(y=predicted,x=switch_urbain_adult,ymin=conf.low,ymax=conf.high,shape=switch_urbain_adult))+   geom_pointrange(position = position_dodge(0.3),size=0.8)+
  theme(plot.title = element_text(size=20,hjust = 0.5,face = "bold"),axis.title.y = element_text(size=22),axis.text.y = element_text(size=15),axis.title.x  = element_text(size=20),axis.text.x  = element_text(size=17),legend.title = element_text(size=24),legend.text =  element_text(size=22),panel.background = element_rect(fill = "white", colour = "grey50"),  panel.grid.major = element_line(colour = "grey90"),plot.tag=element_text(size=20))  +
  
  xlab("Switching Urbanity") + ylab("Lifetime Reproductive Success") +
   scale_y_continuous(limits=c(2,8),breaks = seq(2,8,by = 1)) +    
  scale_x_discrete(limits=c("1.same","2.U-R","3.R-U"),labels =  c("Same Urbanity\n(Same conditions)", "Urban to Rural \n(Bad to Good)", "Rural to Urban\n(Good to Bad)"))+
  geom_text(aes(label = .group ), position = position_dodge(width = 0.35),vjust = -1, hjust = 1.5, size = 5 )+ scale_shape_manual("Switching Urbanity",labels = c("Same urbanity (Same conditions)", "Urban to Rural (Bad to Good)","Rural to Urban (Good to Bad)"), values = c("1.same" = 21, "2.U-R" = 24,"3.R-U" = 25)) + 
  labs(tag = "A")

plot(pl.LRS.surb)
```

### Swithing Shore

```
p_LRS_sriv<- ggpredict(mr_LRS_2, terms = c("switch_rive_adult"))


#name changing necessary to do the beautiful plot
colnames(p_LRS_sriv)[colnames(p_LRS_sriv) == "x"] <- "switch_rive_adult"

p_tuk_LRS_sriv <- merge(p_LRS_sriv ,cld.LRS_sriv, by= "switch_rive_adult")


pl.LRS.sriv <- ggplot(p_tuk_LRS_sriv ,aes(y=predicted,x=switch_rive_adult,ymin=conf.low,ymax=conf.high,colour=switch_rive_adult))+   geom_pointrange(position = position_dodge(0.3),size=0.8)+
  theme(plot.title = element_text(size=20,hjust = 0.5,face = "bold"),axis.title.y = element_text(size=22),axis.text.y = element_text(size=15),axis.title.x  = element_text(size=20),axis.text.x  = element_text(size=17),legend.title = element_text(size=24),legend.text =  element_text(size=22),panel.background = element_rect(fill = "white", colour = "grey50"),  panel.grid.major = element_line(colour = "grey90"),plot.tag=element_text(size=20))  +
  xlab("Switching shore") + ylab("Lifetime Reproductive Success") +
   scale_y_continuous(limits=c(2,8),breaks = seq(2,8,by = 1)) +  
  scale_x_discrete(limits=c("1.same","2.N-S","3.S-N"),labels = c("Same shore\n(Same conditions)", "North to South\n(Bad to Good)", "South to North\n(Good to Bad)"))+
  geom_text(aes(label = .group ), position = position_dodge(width = 0.35),vjust = -1, hjust = 1.5, size = 5 ) +
  scale_color_manual("Switching Shore",labels = c("Same shore (Same conditions)", "North to South (Bad to Good)","South to North (Good to Bad)"), values = c("1.same" = "grey", "2.N-S" = "green","3.S-N" = "orange")) +
  scale_fill_manual("Switching Shore",labels = c("Same shore (Same conditions)", "North to South (Bad to Good)","South to North (Good to Bad)"), values = c("1.same" = "grey", "2.N-S" = "green","3.S-N" = "orange")) +
  labs(tag = "B")

plot(pl.LRS.sriv)
```

# 2) Additional Analysis

## Fertile Years

### Model

```
mr_FY_1 <- glmmTMB(fertile.y ~  urb_riv_parishb  +  scale(wavefront) + scale(dist.km_FR) + switch_urbain_adult+ switch_rive_adult + switch_urbain_adult* switch_rive_adult + Period.hogei +(1 | FamilyID) + (1 | yearb) , family = "gaussian",data = Subset.1750)

summary(mr_FY_1)


car::Anova(mr_FY_1, type = 3, test = "Chisq")

mr_FY_2 <- glmmTMB(fertile.y ~  urb_riv_parishb  +  scale(wavefront) + scale(dist.km_FR) + switch_urbain_adult+ switch_rive_adult +  Period.hogei +(1 | FamilyID) + (1 | yearb) , family = "gaussian",data = Subset.1750)

summary(mr_FY_2)


car::Anova(mr_FY_2, type = 3, test = "Chisq")
```

### Tukey test

```
#FY


e.FY_pb <-emmeans(mr_FY_1 , list(pairwise ~ urb_riv_parishb), adjust = "tukey")
cld.FY_pb<-cld(e.FY_pb , Letter="abcdefg")

e.FY_surb <-emmeans(mr_FY_2 , list(pairwise ~ switch_urbain_adult), adjust = "tukey")
cld.FY_surb<-cld(e.FY_surb , Letter="abcdefg")

e.FY_sriv <-emmeans(mr_FY_2 , list(pairwise ~ switch_rive_adult), adjust = "tukey")
cld.FY_sriv<-cld(e.FY_sriv , Letter="abcdefg")
```

### Birth

```
p_FY_pb <- ggpredict(mr_FY_1, terms = c("urb_riv_parishb"))

p_FY_pb$var <- "Environment of birth"

p_FY_pb[c('Urbanity', 'Shore')] <- str_split_fixed(p_FY_pb$x, '_', 2)

p_tuk_FY_pb <- merge(p_FY_pb ,cld.FY_pb, by.x= "x" , by.y= "urb_riv_parishb")

pl.FY.pb <- ggplot(p_tuk_FY_pb,aes(y=predicted,x=Urbanity,ymin=conf.low,ymax=conf.high,color=Shore))+   geom_pointrange(position = position_dodge(0.3),size=0.8) +
       scale_color_manual("Shore of birth",labels = c("South (Good)", "North (Bad)"), values = c("1.South" = "blue", "2.North" = "red")) +
  theme(plot.title = element_text(size=20,hjust = 0.5,face = "bold"),axis.title.y = element_text(size=22),axis.text.y = element_text(size=15),axis.title.x  = element_text(size=20),axis.text.x  = element_text(size=17),legend.title = element_text(size=22),legend.text =  element_text(size=20),panel.background = element_rect(fill = "white", colour = "grey50"),  panel.grid.major = element_line(colour = "grey90"),plot.tag=element_text(size=20))  +
  xlab("Urbanity of birth") +
  ylab("Fertile Years") +
   scale_y_continuous(limits=c(17,24),breaks = seq(17,24,by = 1)) +   
  scale_x_discrete(limits=c("1.Rural","2.Urban"),labels =  c("Rural\n(Good)", "Urban\n(Bad)"))+
  geom_text(aes(label = .group ), position = position_dodge(width = 0.35),vjust = -1, hjust = 1.2, size = 5 ) +
  labs(tag = "B")

plot(pl.FY.pb)
```

### Swithing Urbanity

```
p_FY_surb<- ggpredict(mr_FY_2, terms = c("switch_urbain_adult"))


#name changing necessary to do the beautiful plot
colnames(p_FY_surb)[colnames(p_FY_surb) == "x"] <- "switch_urbain_adult"

p_tuk_FY_surb <- merge(p_FY_surb ,cld.FY_surb, by= "switch_urbain_adult")


pl.FY.surb <- ggplot(p_tuk_FY_surb, aes(y = predicted, x = switch_urbain_adult, ymin = conf.low, ymax = conf.high, shape = switch_urbain_adult)) +
  geom_pointrange(position = position_dodge(0.3), size = 0.8) +theme(
    plot.title = element_text(size = 20, hjust = 0.5, face = "bold"),
    axis.title.y = element_text(size = 22),
    axis.text.y = element_text(size = 15),
    axis.title.x = element_text(size = 20),
    axis.text.x = element_text(size = 17),
    legend.position = "none",  # Remove the legend
    panel.background = element_rect(fill = "white", colour = "grey50"),
    panel.grid.major = element_line(colour = "grey90"),
    plot.tag = element_text(size = 20))+
  xlab("Switching Urbanity") + ylab("Fertile Years") +
  scale_y_continuous(limits = c(17, 24), breaks = seq(17, 24, by = 1)) +
  scale_x_discrete(limits = c("1.same", "2.U-R", "3.R-U"), labels = c("Same Urbanity\n(Same conditions)", "Urban to Rural \n(Bad to Good)", "Rural to Urban\n(Good to Bad)")) +
  geom_text(aes(label = .group), position = position_dodge(width = 0.35), vjust = -1, hjust = 1.5, size = 5) +
  scale_shape_manual("Switching Urbanity", labels = c("Same urbanity (Same conditions)", "Urban to Rural (Bad to Good)", "Rural to Urban (Good to Bad)"), values = c("1.same" = 21, "2.U-R" = 24, "3.R-U" = 25)) +
  labs(tag = "C") +
  guides(shape = FALSE)


plot(pl.FY.surb)
```

### Swithing Shore

```
p_FY_sriv<- ggpredict(mr_FY_2, terms = c("switch_rive_adult"))


#name changing necessary to do the beautiful plot
colnames(p_FY_sriv)[colnames(p_FY_sriv) == "x"] <- "switch_rive_adult"

p_tuk_FY_sriv <- merge(p_FY_sriv ,cld.FY_sriv, by= "switch_rive_adult")
pl.FY.sriv <- ggplot(p_tuk_FY_sriv, aes(y = predicted, x = switch_rive_adult, ymin = conf.low, ymax = conf.high, colour = switch_rive_adult)) +
  geom_pointrange(position = position_dodge(0.3), size = 0.8) +theme(
    plot.title = element_text(size = 20, hjust = 0.5, face = "bold"),
    axis.title.y = element_text(size = 22),
    axis.text.y = element_text(size = 15),
    axis.title.x = element_text(size = 20),
    axis.text.x = element_text(size = 17),
    legend.position = "none",  # Remove the legend
    panel.background = element_rect(fill = "white", colour = "grey50"),
    panel.grid.major = element_line(colour = "grey90"),
    plot.tag = element_text(size = 20))+
 
  xlab("Switching shore") + ylab("Fertile Years") +
  scale_y_continuous(limits = c(17, 24), breaks = seq(17, 24, by = 1)) +
  scale_x_discrete(
    limits = c("1.same", "2.N-S", "3.S-N"),
    labels = c("Same shore\n(Same conditions)", "North to South\n(Bad to Good)", "South to North\n(Good to Bad)")
  ) +
  geom_text(aes(label = .group), position = position_dodge(width = 0.35), vjust = -1, hjust = 1.5, size = 5) +
  scale_color_manual(
    "Switching Shore",
    labels = c("Same shore (Same conditions)", "North to South (Bad to Good)", "South to North (Good to Bad)"),
    values = c("1.same" = "grey", "2.N-S" = "green", "3.S-N" = "orange")
  ) +
  scale_fill_manual(
    "Switching Shore",
    labels = c("Same shore (Same conditions)", "North to South (Bad to Good)", "South to North (Good to Bad)"),
    values = c("1.same" = "grey", "2.N-S" = "green", "3.S-N" = "orange")
  ) +
  labs(tag = "D")


plot(pl.FY.sriv)
```

## Proportion between NO and LRS

### Model

```
mr_cb_1 <- glmmTMB(cbind(LRS.alt,LRF.alt)~  urb_riv_parishb  +  scale(wavefront) + scale(fertile.y) + scale(dist.km_FR) + switch_urbain_adult+ switch_rive_adult + switch_urbain_adult* switch_rive_adult + Period.hogei + (1 | FamilyID) + (1 | yearb) , family = betabinomial(link = "logit"),data = Subset.1750)

summary(mr_cb_1)


car::Anova(mr_cb_1, type = 3, test = "Chisq")

mr_cb_2 <- glmmTMB(cbind(LRS.alt,LRF.alt)~  urb_riv_parishb  +  scale(wavefront) + scale(fertile.y) + scale(dist.km_FR) + switch_urbain_adult+ switch_rive_adult  + Period.hogei + (1 | FamilyID) + (1 | yearb) , family = betabinomial(link = "logit"),data = Subset.1750)

summary(mr_cb_2)
car::Anova(mr_cb_2, type = 3, test = "Chisq")
```

### Tukey test

```
#cb


e.cb_pb <-emmeans(mr_cb_1, list(pairwise ~ urb_riv_parishb), adjust = "tukey")
cld.cb_pb<-cld(e.cb_pb , Letter="abcdefg")

e.cb_surb <-emmeans(mr_cb_2, list(pairwise ~ switch_urbain_adult), adjust = "tukey")
cld.cb_surb<-cld(e.cb_surb , Letter="abcdefg")

e.cb_sriv <-emmeans(mr_cb_2, list(pairwise ~ switch_rive_adult), adjust = "tukey")
cld.cb_sriv<-cld(e.cb_sriv , Letter="abcdefg")

e.cb_int <-emmeans(mr_cb_1  , list(pairwise ~ switch_urbain_adult* switch_rive_adult), adjust = "tukey")
cld.cb_int<-cld(e.cb_int , Letter="abcdefg")
```

### Birth

```
p_cb_pb <- ggpredict(mr_cb_1, terms = c("urb_riv_parishb"))

p_cb_pb$var <- "Environment of birth"

p_cb_pb[c('Urbanity', 'Shore')] <- str_split_fixed(p_cb_pb$x, '_', 2)

p_tuk_cb_pb <- merge(p_cb_pb ,cld.cb_pb, by.x= "x" , by.y= "urb_riv_parishb")

pl.cb.pb <- ggplot(p_tuk_cb_pb,aes(y=predicted,x=Urbanity,ymin=conf.low,ymax=conf.high,color=Shore))+   geom_pointrange(position = position_dodge(0.3),size=0.8) +
       scale_color_manual("Shore of birth",labels = c("South (Good)", "North (Bad)"), values = c("1.South" = "blue", "2.North" = "red")) +
  theme(plot.title = element_text(size=20,hjust = 0.5,face = "bold"),axis.title.y = element_text(size=22),axis.text.y = element_text(size=15),axis.title.x  = element_text(size=20),axis.text.x  = element_text(size=17),legend.title = element_text(size=22),legend.text =  element_text(size=20),panel.background = element_rect(fill = "white", colour = "grey50"),  panel.grid.major = element_line(colour = "grey90"),plot.tag=element_text(size=20))  +
  xlab("Urbanity of birth") +
  ylab("Proportion between LRS and NO") +
   scale_y_continuous(limits=c(0,1),breaks = seq(0,1,by = 0.2)) +
  scale_x_discrete(limits=c("1.Rural","2.Urban"),labels =  c("Rural\n(Good)", "Urban\n(Bad)"))+
  geom_text(aes(label = .group ), position = position_dodge(width = 0.35),vjust = -1, hjust = 1.2, size = 5 ) +
  labs(tag = "C")

plot(pl.cb.pb)
```

### Swithing Urbanity

```
p_cb_surb<- ggpredict(mr_cb_2, terms = c("switch_urbain_adult"))


#name changing necessary to do the beautiful plot
colnames(p_cb_surb)[colnames(p_cb_surb) == "x"] <- "switch_urbain_adult"

p_tuk_cb_surb <- merge(p_cb_surb ,cld.cb_surb, by= "switch_urbain_adult")


pl.cb.surb <- ggplot(p_tuk_cb_surb ,aes(y=predicted,x=switch_urbain_adult,ymin=conf.low,ymax=conf.high,shape=switch_urbain_adult))+   geom_pointrange(position = position_dodge(0.3),size=0.8) +
  theme(plot.title = element_text(size=20,hjust = 0.5,face = "bold"),axis.title.y = element_text(size=22),axis.text.y = element_text(size=15),axis.title.x  = element_text(size=20),axis.text.x  = element_text(size=17),legend.title = element_text(size=24),legend.text =  element_text(size=22),panel.background = element_rect(fill = "white", colour = "grey50"),  panel.grid.major = element_line(colour = "grey90"),plot.tag=element_text(size=20))  +
  xlab("Switching Urbanity") + ylab("Proportion between NO and LRS") +
         scale_y_continuous(limits=c(0,1),breaks = seq(0,1,by = 0.2)) +
  scale_x_discrete(limits=c("1.same","2.U-R","3.R-U"),labels =  c("Same Urbanity\n(Same conditions)", "Urban to Rural \n(Bad to Good)", "Rural to Urban\n(Good to Bad)"))+
  geom_text(aes(label = .group ), position = position_dodge(width = 0.35),vjust = -1, hjust = 1.5, size = 5 )+ scale_shape_manual("Switching Urbanity",labels = c("Same urbanity (Same conditions)", "Urban to Rural (Bad to Good)","Rural to Urban (Good to Bad)"), values = c("1.same" = 21, "2.U-R" = 24,"3.R-U" = 25)) + 
  labs(tag = "C")

plot(pl.cb.surb)
```

### Swithing Shore

```
p_cb_sriv<- ggpredict(mr_cb_2, terms = c("switch_rive_adult"))


#name changing necessary to do the beautiful plot
colnames(p_cb_sriv)[colnames(p_cb_sriv) == "x"] <- "switch_rive_adult"

p_tuk_cb_sriv <- merge(p_cb_sriv ,cld.cb_sriv, by= "switch_rive_adult")


pl.cb.sriv <- ggplot(p_tuk_cb_sriv ,aes(y=predicted,x=switch_rive_adult,ymin=conf.low,ymax=conf.high,colour=switch_rive_adult))+   geom_pointrange(position = position_dodge(0.3),size=0.8) +
  theme(plot.title = element_text(size=20,hjust = 0.5,face = "bold"),axis.title.y = element_text(size=22),axis.text.y = element_text(size=15),axis.title.x  = element_text(size=20),axis.text.x  = element_text(size=17),legend.title = element_text(size=24),legend.text =  element_text(size=22),panel.background = element_rect(fill = "white", colour = "grey50"),  panel.grid.major = element_line(colour = "grey90"),plot.tag=element_text(size=20))  +
  xlab("Switching shore") + ylab("Proportion between NO and LRS") +
   scale_y_continuous(limits=c(0,1),breaks = seq(0,1,by = 0.2)) +
  scale_x_discrete(limits=c("1.same","2.N-S","3.S-N"),labels = c("Same shore\n(Same conditions)", "North to South\n(Bad to Good)", "South to North\n(Good to Bad)"))+
  geom_text(aes(label = .group ), position = position_dodge(width = 0.35),vjust = -1, hjust = 1.5, size = 5 ) +
  scale_color_manual("Switching Shore",labels = c("Same shore (Same conditions)", "North to South (Bad to Good)","South to North (Good to Bad)"), values = c("1.same" = "grey", "2.N-S" = "green","3.S-N" = "orange")) +
  scale_fill_manual("Switching Shore",labels = c("Same shore (Same conditions)", "North to South (Bad to Good)","South to North (Good to Bad)"), values = c("1.same" = "grey", "2.N-S" = "green","3.S-N" = "orange")) +
  labs(tag = "F")

plot(pl.cb.sriv)
```

### Distance

```
p_cb_dist <- ggpredict(mr_cb_1, terms = "dist.km_FR [all]")
pl.cb.dist <-ggplot(p_cb_dist, aes(x, predicted)) +
  geom_line(colour="blue") +
  geom_ribbon(aes(ymin = conf.low, ymax = conf.high), alpha = .1)+
  theme(plot.title = element_text(size=20,hjust = 0.5,face = "bold"),axis.title.y = element_text(size=22),axis.text.y = element_text(size=12),axis.title.x  = element_text(size=20),axis.text.x  = element_text(size=17),legend.title = element_text(size=22),legend.text =  element_text(size=20),panel.background = element_rect(fill = "white", colour = "grey50"),  panel.grid.major = element_line(colour = "grey90"),plot.tag=element_text(size=20)) +       scale_y_continuous(limits=c(0,1),breaks = seq(0,1,by = 0.2)) +
   xlab("Distance (Km)") + ylab("Proportion between LRS and NO")+
  labs(tag = "B")

plot(pl.cb.dist)
```

### Interaction Switching

```
#prediction of the two variables for switching (Swithing urbanity and switching shore)

p_cb_int<- ggpredict(mr_cb_1 , terms = c("switch_urbain_adult","switch_rive_adult"))


#name changing necessary to do the beautiful plot
colnames(p_cb_int)[colnames(p_cb_int) == "x"]  <- "switch_urbain_adult"
colnames(p_cb_int)[colnames(p_cb_int) == "group"] <- "switch_rive_adult"

p_tuk_cb_int <- merge(p_cb_int ,cld.cb_int, by=c("switch_urbain_adult", "switch_rive_adult"))

#beautiful grey, orange and green plot
pl.cb.int <- ggplot(p_tuk_cb_int,aes(y=predicted,x=switch_urbain_adult,ymin=conf.low,ymax=conf.high,colour=switch_rive_adult,fill=switch_rive_adult,shape=switch_urbain_adult))+   geom_pointrange(position = position_dodge(0.3),size=0.8)+ scale_shape_manual("Switching Urbanity",labels = c("Same urbanity (Same conditions)", "Urban to Rural (Bad to Good)","Rural to Urban (Good to Bad)"), values = c("1.same" = 21, "2.U-R" = 24,"3.R-U" = 25)) +
  scale_color_manual("Switching Shore",labels = c("Same shore (Same conditions)", "North to South (Bad to Good)","South to North (Good to Bad)"), values = c("1.same" = "grey", "2.N-S" = "green","3.S-N" = "orange")) +
  scale_fill_manual("Switching Shore",labels = c("Same shore (Same conditions)", "North to South (Bad to Good)","South to North (Good to Bad)"), values = c("1.same" = "grey", "2.N-S" = "green","3.S-N" = "orange")) + #scale_shape_manual(name  = "Switching Urbanity", breaks=c("1.same", "2.U-R","3.R-U"),labels=c("1.same"="Same urbanity (Same conditions)", "2.U-R"="Urban to Rural (Bad to Good)","3.R-U"="Rural to Urban (Good to Bad)"),values=c("1.same"=21,"2.U-R"=24,"3.R-U"=25))+
  theme(
    plot.title = element_text(size = 20, hjust = 0.5, face = "bold"),
    axis.title.y = element_text(size = 22),
    axis.text.y = element_text(size = 15),
    axis.title.x = element_text(size = 20),
    axis.text.x = element_text(size = 17),
    legend.position = "right",  # Remove the legend
    legend.title = element_text(size = 25),  # Increase legend title size
    legend.text = element_text(size = 22),  # Increase legend text size
    legend.key.size = unit(3, 'lines'),  # Increase size of legend keys
    legend.key.height = unit(3, 'lines'),  # Increase height of legend keys
    legend.key.width = unit(3, 'lines'),  # Increase width of legend keys
    panel.background = element_rect(fill = "white", colour = "grey50"),
    panel.grid.major = element_line(colour = "grey90"),
    plot.tag = element_text(size = 20))+
  xlab("Switching Urbanity") + ylab("Proportion between NO and LRS") +
 scale_y_continuous(limits=c(0,1),breaks = seq(0,1,by = 0.1)) +  
  scale_x_discrete(limits=c("1.same","2.U-R","3.R-U"),labels =  c("Same Urbanity\n(Same conditions)", "Urban to Rural\n(Bad to Good)", "Rural to Urban\n(Good to Bad)"))+
  geom_text(aes(label = .group ), position = position_dodge(width = 0.35),vjust = -0.8, hjust = 0.5, size = 5 ) +
  labs(tag = "E") 

plot(pl.cb.int)
```

# 3) Sensitivity Analysis

## Age at Marriage

### Model

```
mr_agem_1 <- glmmTMB(agem~  +urb_riv_parishb  +  scale(wavefront) + scale(dist.km_FR) + switch_urbain_adult+ switch_rive_adult + switch_urbain_adult* switch_rive_adult+ Period.hogei + (1 | FamilyID) + (1 | yearb) , family = "gaussian",data = Subset.1750)

summary(mr_agem_1)

car::Anova(mr_agem_1, type = 3, test = "Chisq")


mr_agem_2 <- glmmTMB(agem~  +urb_riv_parishb  +  scale(wavefront) + scale(dist.km_FR) + switch_urbain_adult+ switch_rive_adult + Period.hogei + (1 | FamilyID) + (1 | yearb) , family = "gaussian",data = Subset.1750)

summary(mr_agem_2)
```

### Tukey test

```
#agem


e.agem_pb <-emmeans(mr_agem_1  , list(pairwise ~ urb_riv_parishb), adjust = "tukey")
cld.agem_pb<-cld(e.agem_pb , Letter="abcdefg")

e.agem_surb <-emmeans(mr_agem_2  , list(pairwise ~ switch_urbain_adult), adjust = "tukey")
cld.agem_surb<-cld(e.agem_surb , Letter="abcdefg")

e.agem_sriv <-emmeans(mr_agem_2  , list(pairwise ~ switch_rive_adult), adjust = "tukey")
cld.agem_sriv<-cld(e.agem_sriv , Letter="abcdefg")

e.agem_int <-emmeans(mr_agem_1  , list(pairwise ~ switch_urbain_adult* switch_rive_adult), adjust = "tukey")
cld.agem_int<-cld(e.agem_int , Letter="abcdefg")
```

### Birth

```
p_agem_pb <- ggpredict(mr_agem_1 , terms = c("urb_riv_parishb"))

p_agem_pb$var <- "Environment of birth"

p_agem_pb[c('Urbanity', 'Shore')] <- str_split_fixed(p_agem_pb$x, '_', 2)

p_tuk_agem_pb <- merge(p_agem_pb ,cld.agem_pb, by.x= "x" , by.y= "urb_riv_parishb")

pl.agem.pb <- ggplot(p_tuk_agem_pb,aes(y=predicted,x=Urbanity,ymin=conf.low,ymax=conf.high,color=Shore))+   geom_pointrange(position = position_dodge(0.3),size=0.8) +
       scale_color_manual("Shore of birth",labels = c("South (Good)", "North (Bad)"), values = c("1.South" = "blue", "2.North" = "red")) + #to add the colors and the legend of the colors
  
  theme(plot.title = element_text(size=20,hjust = 0.5,face = "bold"),axis.title.y = element_text(size=22),axis.text.y = element_text(size=12),axis.title.x  = element_text(size=20),axis.text.x  = element_text(size=14),legend.title = element_text(size=16),legend.text =  element_text(size=14),panel.background = element_rect(fill = "white", colour = "grey50"),  panel.grid.major = element_line(colour = "grey90"),plot.tag=element_text(size=20)) +
  xlab("Urbanity of birth") +
  ylab("Age at Marriage") +
 scale_y_continuous(limits=c(15.5,26),breaks = seq(16,26,by = 1)) + 
  scale_x_discrete(limits=c("1.Rural","2.Urban"),labels =  c("Rural\n(Good)", "Urban\n(Bad)"))+
  geom_text(aes(label = .group ), position = position_dodge(width = 0.35),vjust = -1.4, hjust = 0.8, size = 5 )+
  labs(tag = "A") 

plot(pl.agem.pb)
```

### Interaction Switching

```
#prediction of the two variables for switching (Swithing urbanity and switching shore)

p_agem_int<- ggpredict(mr_agem_1 , terms = c("switch_urbain_adult","switch_rive_adult"))


#name changing necessary to do the beautiful plot
colnames(p_agem_int)[colnames(p_agem_int) == "x"]  <- "switch_urbain_adult"
colnames(p_agem_int)[colnames(p_agem_int) == "group"] <- "switch_rive_adult"

p_tuk_agem_int <- merge(p_agem_int ,cld.agem_int, by=c("switch_urbain_adult", "switch_rive_adult"))

#beautiful grey, orange and green plot
pl.agem.int <- ggplot(p_tuk_agem_int,aes(y=predicted,x=switch_urbain_adult,ymin=conf.low,ymax=conf.high,colour=switch_rive_adult,fill=switch_rive_adult,shape=switch_urbain_adult))+   geom_pointrange(position = position_dodge(0.3),size=0.8)+ scale_shape_manual("Switching Urbanity",labels = c("Same urbanity (Same conditions)", "Urban to Rural (Bad to Good)","Rural to Urban (Good to Bad)"), values = c("1.same" = 21, "2.U-R" = 24,"3.R-U" = 25)) +
  scale_color_manual("Switching Shore",labels = c("Same shore (Same conditions)", "North to South (Bad to Good)","South to North (Good to Bad)"), values = c("1.same" = "grey", "2.N-S" = "green","3.S-N" = "orange")) +
  scale_fill_manual("Switching Shore",labels = c("Same shore (Same conditions)", "North to South (Bad to Good)","South to North (Good to Bad)"), values = c("1.same" = "grey", "2.N-S" = "green","3.S-N" = "orange")) + #scale_shape_manual(name  = "Switching Urbanity", breaks=c("1.same", "2.U-R","3.R-U"),labels=c("1.same"="Same urbanity (Same conditions)", "2.U-R"="Urban to Rural (Bad to Good)","3.R-U"="Rural to Urban (Good to Bad)"),values=c("1.same"=21,"2.U-R"=24,"3.R-U"=25))+
  
  theme(plot.title = element_text(size=20,hjust = 0.5,face = "bold"),axis.title.y = element_text(size=22),axis.text.y = element_text(size=12),axis.title.x  = element_text(size=20),axis.text.x  = element_text(size=14),legend.title = element_text(size=16),legend.text =  element_text(size=14),panel.background = element_rect(fill = "white", colour = "grey50"),  panel.grid.major = element_line(colour = "grey90"),plot.tag=element_text(size=20)) +
  xlab("Switching Urbanity") + ylab("Age at Marriage") +
 scale_y_continuous(limits=c(15.5,26),breaks = seq(16,26,by = 1)) + 
  scale_x_discrete(limits=c("1.same","2.U-R","3.R-U"),labels =  c("Same Urbanity\n(Same conditions)", "Urban to Rural\n(Bad to Good)", "Rural to Urban\n(Good to Bad)"))+
  geom_text(aes(label = .group ), position = position_dodge(width = 0.35),vjust = -0.8, hjust = 0.5, size = 5 ) +
  labs(tag = "B") 

plot(pl.agem.int)
```

### Distance

```
p_agem_dist <- ggpredict(mr_agem_1 , terms = "dist.km_FR [all]")
pl.agem.dist <-ggplot(p_agem_dist, aes(x, predicted)) +
  geom_line(colour="blue") +
  geom_ribbon(aes(ymin = conf.low, ymax = conf.high), alpha = .1)+
  theme(plot.title = element_text(size=20,hjust = 0.5,face = "bold"),axis.title.y = element_text(size=22),axis.text.y = element_text(size=12),axis.title.x  = element_text(size=20),axis.text.x  = element_text(size=17),legend.title = element_text(size=22),legend.text =  element_text(size=20),panel.background = element_rect(fill = "white", colour = "grey50"),  panel.grid.major = element_line(colour = "grey90"),plot.tag=element_text(size=20)) +    scale_y_continuous(limits=c(15.5,26),breaks = seq(16,26,by = 1)) +  
   xlab("Distance (Km)") + ylab("Age at Marriage")+
  labs(tag = "C")

plot(pl.agem.dist)
```

# 4) Merged plots

## Main Manuscript

### Birth (Fig.1)

```
library(patchwork)

pl.Env_birth <-pl.AFR.pb  / pl.LRS.pb + plot_layout(guides = "collect",nrow = 1, ncol=2)+ plot_annotation(
    theme = theme(plot.title = element_text(size = 26,hjust=0.5),legend.title = element_text(size=22),legend.text =  element_text(size=20)),
  
)

plot(pl.Env_birth)
```

```
ggsave("Figure1.tiff",width=12, height=6, dpi=600)
```

### Adult (Fig.3)

```
pl.adult <-  pl.LRS.surb +   pl.LRS.sriv   + plot_layout(guides = "collect",nrow = 1, ncol=2)+ 
  plot_annotation(
    theme = theme(plot.title = element_text(size = 26,hjust=0.5),legend.title = element_text(size=22),legend.text =  element_text(size=20)),)

plot(pl.adult)
```

```
ggsave("Figure3.tiff",width=18, height=6, dpi=400)
```

## Supplementary Material

### Additional analysis

#### Birth (Fig.S3)

```
pl.Env_birth.sup <-  pl.NO.pb +pl.FY.pb + pl.cb.pb +  plot_layout(guides = "collect", ncol=3)+ plot_annotation(
    theme = theme(plot.title = element_text(size = 26,hjust=0.5),legend.title = element_text(size=22),legend.text =  element_text(size=20)),
)

plot(pl.Env_birth.sup )
```

```
ggsave("FigureS3.tiff",width=15, height=6, dpi=600)
```

#### Adult (Fig.S4)

```
library(patchwork)

# Diseñamos el layout
design <- "
ABE
CDE
"

# Construimos el plot con el diseño especificado
pl.adult.additional <- (pl.NO.surb + pl.FY.surb + 
                        pl.NO.sriv + pl.FY.sriv) +
                       pl.cb.int +
                       plot_layout(design = design) + 
                       plot_annotation(
                         theme = theme(
                           plot.title = element_text(size = 26, hjust = 0.5),
                           legend.title = element_text(size = 30),
                           legend.text = element_text(size = 30), legend.position = "right"
                         ))
                       

# Mostramos el plot
plot(pl.adult.additional)
```

```
# Guardamos la figura
ggsave("FigureS4.tiff", plot = pl.adult.additional, width = 22, height = 10, dpi = 400)
```

```
# Install cowplot if necessary
# install.packages("cowplot")

library(ggplot2)
library(patchwork)
library(cowplot)

# Extract the legend from pl.cb.int
legend_cb_int <- get_legend(pl.cb.int)

# Define the layout
design <- "
ABE
CDE
"

# Create an empty plot with just the legend
legend_plot <- ggdraw() + draw_grob(legend_cb_int)

# Construct the combined plot with the legend
pl.adult.additional <- (pl.NO.surb + pl.FY.surb + 
                        pl.NO.sriv + pl.FY.sriv + pl.cb.int +legend_plot) +
                       plot_layout(design = design) + 
                       plot_annotation(
                         theme = theme(
                           plot.title = element_text(size = 26, hjust = 0.5),
                           legend.title = element_text(size = 28),
                           legend.text = element_text(size = 25)
                         ))

# Display the combined plot
plot(pl.adult.additional)
```

```
# Save the figure
ggsave("FigureS4.tiff", plot = pl.adult.additional, width = 22, height = 10, dpi = 400)
```

### Sensitivity analysis (Fig.S5)

```
pl.Sensitivity <- pl.agem.pb + pl.agem.int + 
  plot_layout(widths = c(1, 2))

ggsave("FigureS5.tiff",width=15, height=6, dpi=600)
```

### Distance (Fig.S6)

```
pl.distance <- pl.AFR.dist  +pl.cb.dist + pl.agem.dist + plot_layout(guides = "collect")


ggsave("FigureS6.tiff",width=12, height=6, dpi=600)
```

### Vs (Fig.S7)

```
AFRvsNO <- ggplot(data=Subset.1750, aes(x=AFR, y=NO)) +
    geom_point() + geom_smooth(method = lm) + labs(title = "NO Vs. AFR",
    x = "Age at First Reproduction", y = "Number of Offspring")+   theme(plot.title = element_text(size=20,hjust = 0.5,face = "bold"),axis.title.y = element_text(size=22),axis.text.y = element_text(size=12),axis.title.x  = element_text(size=20),axis.text.x  = element_text(size=17),legend.title = element_text(size=22),legend.text =  element_text(size=20),panel.background = element_rect(fill = "white", colour = "grey50"),  panel.grid.major = element_line(colour = "grey90"),plot.tag=element_text(size=20))  +
    scale_y_continuous(limits=c(0,23),breaks = seq(0,23,by = 2))+
    scale_x_continuous(limits=c(12,47),breaks = seq(15,45,by = 5))+
  labs(tag = "A")


AFRvsLRS <- ggplot(data=Subset.1750, aes(x=AFR, y=LRS.alt)) +
    geom_point() + geom_smooth(method = lm) + labs(title = "LRS Vs. AFR",
    x = "Age at First Reproduction", y = "Lifetime Reproductive Success")+   theme(plot.title = element_text(size=20,hjust = 0.5,face = "bold"),axis.title.y = element_text(size=22),axis.text.y = element_text(size=12),axis.title.x  = element_text(size=20),axis.text.x  = element_text(size=17),legend.title = element_text(size=22),legend.text =  element_text(size=20),panel.background = element_rect(fill = "white", colour = "grey50"),  panel.grid.major = element_line(colour = "grey90"),plot.tag=element_text(size=20))  +
    scale_y_continuous(limits=c(0,23),breaks = seq(0,23,by = 2))+
    scale_x_continuous(limits=c(12,47),breaks = seq(15,45,by = 5))+
  labs(tag = "B")


pl.VS <- AFRvsNO   +AFRvsLRS +  plot_layout(guides = "collect")


FYvsNO <- ggplot(data=Subset.1750, aes(x=fertile.y, y=NO)) +
    geom_point() + geom_smooth(method = lm) + labs(title = "NO Vs. Fertile Years",
    x = "Fertile Years", y = "Number of Offspring")+   theme(plot.title = element_text(size=20,hjust = 0.5,face = "bold"),axis.title.y = element_text(size=22),axis.text.y = element_text(size=12),axis.title.x  = element_text(size=20),axis.text.x  = element_text(size=17),legend.title = element_text(size=22),legend.text =  element_text(size=20),panel.background = element_rect(fill = "white", colour = "grey50"),  panel.grid.major = element_line(colour = "grey90"),plot.tag=element_text(size=20))  +
    scale_y_continuous(limits=c(0,23),breaks = seq(0,23,by = 2))+
    scale_x_continuous(limits=c(0,35),breaks = seq(0,35,by = 5))+
  labs(tag = "C")


FYvsLRS <- ggplot(data=Subset.1750, aes(x=fertile.y, y=LRS.alt)) +
    geom_point() + geom_smooth(method = lm) + labs(title = "LRS Vs. Fertile Years",
    x = "Fertile Years", y = "Lifetime Reproductive Success")+   theme(plot.title = element_text(size=20,hjust = 0.5,face = "bold"),axis.title.y = element_text(size=22),axis.text.y = element_text(size=12),axis.title.x  = element_text(size=20),axis.text.x  = element_text(size=17),legend.title = element_text(size=22),legend.text =  element_text(size=20),panel.background = element_rect(fill = "white", colour = "grey50"),  panel.grid.major = element_line(colour = "grey90"),plot.tag=element_text(size=20))  +
    scale_y_continuous(limits=c(0,23),breaks = seq(0,23,by = 2))+
    scale_x_continuous(limits=c(0,35),breaks = seq(0,35,by = 5))+
  labs(tag = "D")


pl.VS <- AFRvsNO   +AFRvsLRS + FYvsNO   +FYvsLRS +  plot_layout(guides = "collect")


ggsave("FigureS7.tiff",width=12, height=12, dpi=400)
```
