# Supplementary material for: Early and adult life environmental effects on reproductive performance in preindustrial women
Source: PLoS One. 2024 Oct 28;19(10):e0290212. doi: 10.1371/journal.pone.0290212 (PMC11515999; doi:10.1371/journal.pone.0290212)
Supplement: S4 Data — (HTML) [file pone.0290212.s025.html]

Sequential models


# Sequential models

#### 2024-06-17

Libraries

```
library(glmmTMB)
library(readr)
library(ggplot2)
library(ggeffects)
library(cowplot)
library(ggplot2)
library(ggpubr)
library(dplyr)
library(DHARMa)
library(MuMIn)
library(glmm.hp)
```

The following files are needed

- Subset.1750
- Subset.1729

# AFR

```
ms_I_AFR_1 <-glmmTMB(AFR~  urb_riv_parishb  +  scale(wavefront) +  scale(dist.km_FR) + Period.hogei + (1 | FamilyID) + (1 | yearb) , family = "gaussian",data = Subset.1750)

ms_II_AFR_1 <-glmmTMB(AFR~  urb_riv_adult  +  scale(wavefront) +  scale(dist.km_FR) + Period.hogei + (1 | FamilyID) + (1 | yearb) , family = "gaussian",data = Subset.1750)

ms_III_AFR_1 <-glmmTMB(AFR~   switch_rive_adult +  scale(wavefront) +  scale(dist.km_FR) + Period.hogei + (1 | FamilyID) + (1 | yearb) , family = "gaussian",data = Subset.1750)

ms_IV_AFR_1 <-glmmTMB(AFR~   switch_urbain_adult +  scale(wavefront) +  scale(dist.km_FR) + Period.hogei + (1 | FamilyID) + (1 | yearb) , family = "gaussian",data = Subset.1750)


ms_V_AFR_1 <-glmmTMB(AFR~  urb_riv_parishb  + urb_riv_adult + scale(wavefront) +  scale(dist.km_FR) + Period.hogei + (1 | FamilyID) + (1 | yearb) , family = "gaussian",data = Subset.1750)

ms_VI_AFR_1 <-glmmTMB(AFR~  urb_riv_parishb  + switch_rive_adult +  scale(wavefront) +  scale(dist.km_FR) + Period.hogei + (1 | FamilyID) + (1 | yearb) , family = "gaussian",data = Subset.1750)

ms_VII_AFR_1 <-glmmTMB(AFR~  urb_riv_parishb  + switch_urbain_adult +  scale(wavefront) +  scale(dist.km_FR) + Period.hogei + (1 | FamilyID) + (1 | yearb) , family = "gaussian",data = Subset.1750)

ms_VIII_AFR_1 <-glmmTMB(AFR~  urb_riv_parishb  + switch_urbain_adult* switch_rive_adult +  scale(wavefront) +  scale(dist.km_FR) + Period.hogei + (1 | FamilyID) + (1 | yearb) , family = "gaussian",data = Subset.1750)
```

# NO

```
ms_I_NO_1 <-glmmTMB(NO~  urb_riv_parishb  +  scale(wavefront) + scale(fertile.y) +  scale(dist.km_FR) + Period.hogei + (1 | FamilyID) + (1 | yearb) , family = "poisson",data = Subset.1750)

ms_II_NO_1 <-glmmTMB(NO~  urb_riv_adult  +  scale(wavefront) + scale(fertile.y) +  scale(dist.km_FR) + Period.hogei + (1 | FamilyID) + (1 | yearb) , family = "poisson",data = Subset.1750)

ms_III_NO_1 <-glmmTMB(NO~   switch_rive_adult +  scale(wavefront) + scale(fertile.y) +  scale(dist.km_FR) + Period.hogei + (1 | FamilyID) + (1 | yearb) , family = "poisson",data = Subset.1750)

ms_IV_NO_1 <-glmmTMB(NO~   switch_urbain_adult +  scale(wavefront) + scale(fertile.y) +  scale(dist.km_FR) + Period.hogei + (1 | FamilyID) + (1 | yearb) , family = "poisson",data = Subset.1750)


ms_V_NO_1 <-glmmTMB(NO~  urb_riv_parishb  + urb_riv_adult + scale(wavefront) +  scale(dist.km_FR) + Period.hogei + (1 | FamilyID) + (1 | yearb) , family = "poisson",data = Subset.1750)

ms_VI_NO_1 <-glmmTMB(NO~  urb_riv_parishb  + switch_rive_adult +  scale(wavefront) + scale(fertile.y) +  scale(dist.km_FR) + Period.hogei + (1 | FamilyID) + (1 | yearb) , family = "poisson",data = Subset.1750)

ms_VII_NO_1 <-glmmTMB(NO~  urb_riv_parishb  + switch_urbain_adult +  scale(wavefront) + scale(fertile.y) +  scale(dist.km_FR) + Period.hogei + (1 | FamilyID) + (1 | yearb) , family = "poisson",data = Subset.1750)

ms_VIII_NO_1 <-glmmTMB(NO~  urb_riv_parishb  + switch_urbain_adult* switch_rive_adult +  scale(wavefront) + scale(fertile.y) +  scale(dist.km_FR) + Period.hogei + (1 | FamilyID) + (1 | yearb) , family = "poisson",data = Subset.1750)
```

# LRS

```
ms_I_LRS_1 <-glmmTMB(LRS.alt~  urb_riv_parishb  +  scale(wavefront) + scale(fertile.y) +  scale(dist.km_FR) + Period.hogei + (1 | FamilyID) + (1 | yearb) , family = "poisson",data = Subset.1729)

ms_II_LRS_1 <-glmmTMB(LRS.alt~  urb_riv_adult  +  scale(wavefront) + scale(fertile.y) +  scale(dist.km_FR) + Period.hogei + (1 | FamilyID) + (1 | yearb) , family = "poisson",data = Subset.1729)

ms_III_LRS_1 <-glmmTMB(LRS.alt~   switch_rive_adult +  scale(wavefront) + scale(fertile.y) +  scale(dist.km_FR) + Period.hogei + (1 | FamilyID) + (1 | yearb) , family = "poisson",data = Subset.1729)

ms_IV_LRS_1 <-glmmTMB(LRS.alt~   switch_urbain_adult +  scale(wavefront) + scale(fertile.y) +  scale(dist.km_FR) + Period.hogei + (1 | FamilyID) + (1 | yearb) , family = "poisson",data = Subset.1729)


ms_V_LRS_1 <-glmmTMB(LRS.alt~  urb_riv_parishb  + urb_riv_adult + scale(wavefront) +  scale(dist.km_FR) + Period.hogei + (1 | FamilyID) + (1 | yearb) , family = "poisson",data = Subset.1729)

ms_VI_LRS_1 <-glmmTMB(LRS.alt~  urb_riv_parishb  + switch_rive_adult +  scale(wavefront) + scale(fertile.y) +  scale(dist.km_FR) + Period.hogei + (1 | FamilyID) + (1 | yearb) , family = "poisson",data = Subset.1729)

ms_VII_LRS_1 <-glmmTMB(LRS.alt~  urb_riv_parishb  + switch_urbain_adult +  scale(wavefront) + scale(fertile.y) +  scale(dist.km_FR) + Period.hogei + (1 | FamilyID) + (1 | yearb) , family = "poisson",data = Subset.1729)

ms_VIII_LRS_1 <-glmmTMB(LRS.alt~  urb_riv_parishb  + switch_urbain_adult* switch_rive_adult +  scale(wavefront) + scale(fertile.y) +  scale(dist.km_FR) + Period.hogei + (1 | FamilyID) + (1 | yearb) , family = "poisson",data = Subset.1729)
```

# SUMMARY

```
# Load required libraries
library(officer)
library(flextable)
```

```
## 
## Attaching package: 'flextable'
```

```
## The following objects are masked from 'package:ggpubr':
## 
##     border, font, rotate
```

```
library(glmmTMB)
library(MuMIn) # Load the MuMIn package for R² calculation

# Create a list of models with names
model_list <- list(
  "AFR, Birth Environment" = ms_I_AFR_1,
  "AFR, Adult life environment " = ms_II_AFR_1,
  "AFR, Switching Shore" = ms_III_AFR_1,
  "AFR, Switching Urbanity" = ms_IV_AFR_1,
  "AFR, Birth environment + Adult life environment" = ms_V_AFR_1,
  "AFR, Birth environment + Switching Shore" = ms_VI_AFR_1,
  "AFR, Birth environment + Switching Urbanity" = ms_VII_AFR_1,
  "AFR, Birth environment + Switching Urbanity*Switching Shore" = ms_VIII_AFR_1,
  "NO, Birth Environment" = ms_I_NO_1,
  "NO, Adult life environment " = ms_II_NO_1,
  "NO, Switching Shore" = ms_III_NO_1,
  "NO, Switching Urbanity" = ms_IV_NO_1,
  "NO, Birth environment + Adult life environment" = ms_V_NO_1,
  "NO, Birth environment + Switching Shore" = ms_VI_NO_1,
  "NO, Birth environment + Switching Urbanity" = ms_VII_NO_1,
  "NO, Birth environment + Switching Urbanity*Switching Shore" = ms_VIII_NO_1,
  "LRS, Birth Environment" = ms_I_LRS_1,
  "LRS, Adult life environment " = ms_II_LRS_1,
  "LRS, Switching Shore" = ms_III_LRS_1,
  "LRS, Switching Urbanity" = ms_IV_LRS_1,
  "LRS, Birth environment + Adult life environment" = ms_V_LRS_1,
  "LRS, Birth environment + Switching Shore" = ms_VI_LRS_1,
  "LRS, Birth environment + Switching Urbanity" = ms_VII_LRS_1,
  "LRS, Birth environment + Switching Urbanity*Switching Shore" = ms_VIII_LRS_1
)

# Function to calculate R²
get_r_squared <- function(model) {
  r2 <- tryCatch({
    r.squaredGLMM(model)
  }, error = function(e) {
    return(NULL)
  })
  
  if (!is.null(r2) && ncol(r2) > 1) {
    # Select the marginal and conditional R² and round them to three decimals
    return(data.frame(R2m = round(r2[1, "R2m"], 3), R2c = round(r2[1, "R2c"], 3)))
  } else {
    return(data.frame(R2m = NA, R2c = NA))
  }
}

# Extract R² values for each model
r_squared_list <- lapply(model_list, get_r_squared)

# Combine R² values into a single data frame
r_squared_df <- do.call(rbind, r_squared_list)

# Add model names as a new column to r_squared_df
r_squared_df$model <- names(model_list)

# Reorder columns to make the "model" column the first column
r_squared_df <- r_squared_df[, c("model", "R2m", "R2c")]

# Create a flextable object
ft_r2 <- flextable(r_squared_df)

# Set the default font family and color for flextable
flextable::set_flextable_defaults(
  font.family = "Times New Roman",
  font.color = "black"
)

# Export the flextable to a Word document
doc_r2 <- read_docx()
doc_r2 <- doc_r2 %>%
  body_add_flextable(ft_r2)
doc_r2 <- doc_r2 %>%
  print(target = "model_r2_summary.docx")
```
