# Supplementary material for: Early and adult life environmental effects on reproductive performance in preindustrial women
Source: PLoS One. 2024 Oct 28;19(10):e0290212. doi: 10.1371/journal.pone.0290212 (PMC11515999; doi:10.1371/journal.pone.0290212)
Supplement: Attachment — Submitted filename: PONE-D-23-24394_Review.pdf [file pone.0290212.s026.pdf]

Review of PONE-D-23-24394: Environmental effects on reproductive performance in preindustrial women

I appreciated the opportunity to review the manuscript “Environmental effects on reproductive performance in preindustrial women.” The authors use an historic database of individuals and family histories from the *Registre de la population du Québec Ancien* (RPQA) to study the effects of moving between urban and rural environments in different regions of pre-industrial Montreal.

Given the authors are studying humans, not animal populations, with this analysis, it is not clear that the hypotheses and statistical analyses are appropriate. These limitations prevent drawing definitive conclusions from the analysis. Below I summarize my major comments on the manuscript.

<u>Environmental conditions:</u>

1. The authors use a very crude classification of environmental conditions: rural-south, rural-north, urban-south, and urban-north and make generalizations regarding how “good” or “bad” the conditions are. With such limited information on the actual environmental conditions, it is not clear how much we actually learn from these classifications and the normative statements are not well-defended with evidence.
2. The authors argue that their approach of using fixed locations (the 4 groupings mentioned above) offers advantages over a temporal approach that examines changing conditions over time. I would argue that an approach that allows for both spatial and temporal variation is preferred. The authors include women born between 1640-1729/1750, thus spanning over 100 years. To say that the temporal changes do not matter over such a long period is incorrect. Particularly when trying to say something regarding urban areas, which undoubtedly changed substantially during that period. While the authors include year of birth to control for cohort effects, this does not account for temporal changes that affect all cohorts in the same time period (but across ages). For example, a famine in a given year may affect the age at first birth for younger cohorts and also the lifetime number of children born for all cohorts.
3. It was unclear from the manuscript how the authors are tracking the age and period within their life course fertility at which people move locations, which undoubtedly matters. For example, do we know whether the “switchers” switched before their first birth? If not, then the authors are associating the wrong conditions with that outcome.

<u>Individual-level determinants of fertility and human behavior:</u>

1. The authors ascribe the environmental conditions of a given parish (rural-south, rural-north, urban-south, and urban-north) to all of the individuals that live in that parish. Even if certain areas tended to be better agriculturally than others, not all individuals fared equally well. It is most certainly better to be a wealthy individual in a “bad” environment than a poor person in a “good” environment. While the RPQA data does not include information on individual or household wealth, it would improve the hypotheses and statistical analysis for the authors to take seriously the role of individual-level variation.
2. Relatedly, while the authors are using a human population to make analogous comparisons to animal populations (e.g., red deer, elephants, etc), they are missing the

seminal literature on determinants of fertility (and disentangling individual, social, behavioral, environmental factors), as well as some seminal literature on early life environments. Reviewing this literature would help to inform the hypotheses the authors test in this analysis and ensure that they are appropriate for studying humans rather than animals. For example, in social science and demography fields it is understood that in the early stages of the fertility transition (which typically corresponds to more rural, pre-industrial times).

Almond, Douglas, and Janet Currie. "Killing Me Softly: The Fetal Origins Hypothesis." *Journal of Economic Perspectives* 25, no. 3 (August 1, 2011): 153–72. https://doi.org/10.1257/jep.25.3.153.

Becker, Gary S., and H. Gregg Lewis. "On the Interaction between the Quantity and Quality of Children." *Journal of Political Economy* 81, no. 2, Part 2 (1973): S279–88. https://doi.org/10.1086/260166.

Bongaarts, John. "Does Malnutrition Affect Fecundity? A Summary of Evidence." *Science* 208, no. 4444 (1980): 564–69.

Bongaarts, John. "Modeling the Fertility Impact of the Proximate Determinants: Time for a Tune-Up." *Demographic Research* 33 (September 11, 2015): 535–60. https://doi.org/10.4054/DemRes.2015.33.19.

Bongaarts, John, and Dennis Hodgson. *Fertility Transition in the Developing World*. SpringerBriefs in Population Studies. Cham: Springer International Publishing, 2022. https://doi.org/10.1007/978-3-031-11840-1.

Friedlander, Dov. "Demographic Responses and Population Change." *Demography* 6, no. 4 (November 1, 1969): 359–81. https://doi.org/10.2307/2060083.

Lesthaeghe, Ron. "The Second Demographic Transition: A Concise Overview of Its Development." *Proceedings of the National Academy of Sciences* 111, no. 51 (December 23, 2014): 18112–15. https://doi.org/10.1073/pnas.1420441111.

3. The authors make several normative statements regarding a younger age at first birth and higher number of offspring being good (e.g., 175-176). While these may represent better reproductive performance in animal populations, it is well established in the human health literature that early age at first birth (often defined as 15 years old or younger) is associated with bad maternal and child health outcomes, such as low birthweight, preterm birth, infant mortality, and maternal mortality. In demographic literature, which emphasizes concepts of reproductive autonomy, it is not the total number of children born to a woman that matters but rather whether she is able to attain her desired family size. In many cases, her desired family size is lower than the number of children she has (so more is not unequivocally better), due to complex and intersecting social, cultural, and

economic reasons. Beyond the issue with calling younger age at first birth and higher number of offspring “good”, this framing ignores (in the statistical sense) how these outcomes are in fact correlated, particularly for human populations, which are presumably quite different from red deer, elephants, and other animal populations in ways not acknowledged by the authors. As a result, certain findings are expressed as surprising, when in fact they are consistent with expectation for humans. For example, the authors state (374-379) that given the correlation between younger AFR and higher NO it was surprising that women born in urban (“bad”) parishes didn’t have more children surviving to adulthood (which they define as 15 years of age). Given younger AFR is associated with lower chances of child survival and a well-established reason for large numbers of pregnancies and children in preindustrial, pre-demographic transition times is to protect against child mortality and have a sufficiently large family to work in farms and other household activities.

Statistical Analyses:

1. While the procedure to remove insignificant terms from statistical models is appropriate in some fields and for some analyses, in this case as the authors wish to test competing hypotheses, any deletion of terms prevents their ability to do so in a systematic and comprehensive manner. If the authors have a theory about which variables determine these fertility outcomes, those variables should all be in the model regardless of statistical significance because any lack of statistical significance informs the interpretation and conclusion regarding the hypotheses.
2. To truly test these different hypotheses, I would suggest introducing the early life, later life, and switching environmental variables in sequentially and then using all combinations. This would allow the authors, for example, to see what happens to early life environmental variables once they control for switching or once they control for later life. Because this approach would also involve a large number of statistical tests, it would be important for the authors to account for multiple hypotheses testing to ensure they are not drawing conclusions from type I errors.
3. A more minor comment on the interpretation of results is that the authors should be careful in how they interpret terms on categorical variables and make sure they are always interpreted as relative to the dropped category. For example, as stated in the notes for Table 2 the reference level is rural south for birth environment, which means the coefficients on rural north, urban south, and urban north are all interpreted as relative to rural south. But in 216-218, the authors interpret the coefficients on the urban variables and relative to both rural variables.
