# Supplementary material for: Early and adult life environmental effects on reproductive performance in preindustrial women
Source: PLoS One. 2024 Oct 28;19(10):e0290212. doi: 10.1371/journal.pone.0290212 (PMC11515999; doi:10.1371/journal.pone.0290212)
Supplement: Attachment — Submitted filename: Response to Reviewers.docx [file pone.0290212.s027.docx]

Dear Associate Editor,

Thank you very much for the feedback on our manuscript entitled, “Early and adult life environmental effects on reproductive performance in preindustrial women”. We especially appreciated the comments about better justifying how demographic data on humans could complement wildlife eco-evolutionary questions. In that sense, and among the other comments, we have thoroughly revised our manuscript and believe that the improvements will enhance its interest and value to the readers of PLOS ONE. As you will notice, addressing the comments also required us to improve the flow of the manuscript in areas not necessarily highlighted by the reviewers (for consistency purposes). We, indeed, identified all major modifications with the track change version provided.

Below, we provide a detailed a point-by-point response to the reviewer’s comments, indicated in *italic*s. We have numbered each response consecutively to facilitate cross-referencing.

We thank you in advance for considering our revised submission, and we look forward to hearing from you in due course.

Yours sincerely,

Lidia Colejo Durán, on behalf of all co-authors.

*Review of PONE-D-23-24394: Environmental effects on reproductive performance in preindustrial women*

*I appreciated the opportunity to review the manuscript “Environmental effects on reproductive performance in preindustrial women.” The authors use an historic database of individuals and family histories from the Registre de la population du Québec Ancien (RPQA) to study the effects of moving between urban and rural environments in different regions of pre-industrial Montreal.*

*Given the authors are studying humans, not animal populations, with this analysis, it is not clear that the hypotheses and statistical analyses are appropriate. These limitations prevent drawing definitive conclusions from the analysis. Below I summarize my major comments on the manuscript.*

We thank the review for the time invested in revising our work. We indeed use an eco-evolutionary approach to analyse human data. As previously reported by demographers’, pre-industrial humans lived in natural conditions, experienced natural fertility regime, and thus were subject to ecological forces [1]. Others also argued that well-conducted observational studies on humans across multiple generations can offer valuable insights into key topics in evolutionary ecology [2]. In addition, Lumma and Clutton-Brock suggested that integrating studies of wild animals and humans could enhance our understanding of how early development influences reproductive performance across generations and analyse several examples of long-term negative effects of adverse early development on survival and reproduction in humans [3]. For instance, people born during the annual “hungry season” are up to 10 times more likely to die prematurely in young adulthood [4] and mothers born weighing 2,000 grams or less were at higher risk for poor pregnancy outcomes, despite their babies not being smaller than those of heavier-born mothers [5].

These studies emphasize the importance of quantifying the direction and magnitude of early life environmental effects on life trajectories for a set of traits closely related to fitness [6,7]. Considering the comments, we have now expanded the introduction to integrate studies using similar datasets and shed light into important evolutionary questions [3,8–10]. We have also put more emphasise on the importance of considering evolutionary processes in human demographic and reproductive ecology studies. We have considered all suggestions made by the reviewer in this comment to improve clarity of our hypothesis and analyses. We hope these changes, included between the lines 52–62 have improved our manuscript.

As a note, during the period of Nouvelle-France, there were only two urban areas where people could reside: Montréal and Québec City.

*Environmental conditions:*

*1. The authors use a very crude classification of environmental conditions: rural-south,*

*rural-north, urban-south, and urban-north and make generalizations regarding how*

*“good” or “bad” the conditions are. With such limited information on the actual*

*environmental conditions, it is not clear how much we actually learn from these*

*classifications and the normative statements are not well-defended with evidence.*

We have used broad categories to characterise the environmental conditions. We now acknowledge those limitations in the text. Nonetheless, we are using environment categorisation based on previously described literature, [11–14]. We have now expanded the differences between the environmental characteristics of the north and the south shore of the St Lawrence River in lines 152–165. Additionally, as mentioned in the manuscript in lines 166–173, urban and rural populations exhibited differential mortality rates due to socio-economic factors [15].

*2. The authors argue that their approach of using fixed locations (the 4 groupings mentioned*

*above) offers advantages over a temporal approach that examines changing conditions*

*over time. I would argue that an approach that allows for both spatial and temporal*

*variation is preferred. The authors include women born between 1640–1729/1750, thus*

*spanning over 100 years. To say that the temporal changes do not matter over such a long*

*period is incorrect. Particularly when trying to say something regarding urban areas,*

*which undoubtedly changed substantially during that period. While the authors include*

*year of birth to control for cohort effects, this does not account for temporal changes that*

*affect all cohorts in the same time period (but across ages). For example, a famine in a*

*given year may affect the age at first birth for younger cohorts and also the lifetime*

*number of children born for all cohorts.*

The reviewer is right, both time and space are important and sometimes hard to distinguish. We believe we duly accounted for, if not at least acknowledge those challenges. Previously we controlled for cohort using “*year of birth”* as a random effect and for temporal variation with “*Wave front*”, defined as the time between the foundation of the parish of birth of the mother and the birth of her first offspring (see lines 232–234). However, to further address the comments of the reviewer, we have now redone all analyses with the addition of the variable *Period**,* as has previously been done in the literature [13,16]. The variable *Period* is categorical and describes five intervals, given the date of birth of the woman [8]. The justification is now provided on lines 235–244.

*3. It was unclear from the manuscript how the authors are tracking the age and period*

*within their life course fertility at which people move locations, which undoubtedly matters. For example, do we know whether the “switchers” switched before their first birth? If not, then the authors are associating the wrong conditions with that outcome.*

We agree that our ability to track the movements of the studied individuals is limited to the punctual data obtained from baptism, marriage, and burial registers, focusing on when these events occur to them or close family members. However, we now point out on lines 174–175 that it has been previously done in other papers on the population [11,14].

We also clarified this point in the methods on lines 183–195 and acknowledge the limitations of this approach on lines 394–397 in the discussion, as tracking the full lifetime movement was not possible with the type of data available.

*Individual-level determinants of fertility and human behavior:*

*1. The authors ascribe the environmental conditions of a given parish (rural-south, rural-north, urban-south, and urban-north) to all of the individuals that live in that parish. Even*

*if certain areas tended to be better agriculturally than others, not all individuals fared*

*equally well. It is most certainly better to be a wealthy individual in a “bad” environment*

*than a poor person in a “good” environment. While the RPQA data does not include*

*information on individual or household wealth, it would improve the hypotheses and*

*statistical analysis for the authors to take seriously the role of individual-level variation.*

We completely agree with the reviewer’s comment and acknowledge that we lack data on household wealth (See lines 393–396). We now emphasised that “*Family ID*” was included as a random factor in all our models to control for potential genetic and shared family environment effects. As the Family ID is the marriage ID of the parents of the woman, this would control for families with more wealth or whose farms were more productive, which would impact the health of the woman, along with her marriage perspectives [13,17]. Interestingly, random effects account for more of the variance in AFR compared to NO and LRS. This suggests that family background significantly influences AFR but has a much smaller impact on NO and LRS. We mentioned this in the discussion on lines 390–392 (see also Table S5).

*2. Relatedly, while the authors are using a human population to make analogous*

*comparisons to animal populations (e.g., red deer, elephants, etc), they are missing the seminal literature on determinants of fertility (and disentangling individual, social, behavioral, environmental factors), as well as some seminal literature on early life environments. Reviewing this literature would help to inform the hypotheses the author’s test in this analysis and ensure that they are appropriate for studying humans rather than animals. For example, in social science and demography fields it is understood that in the early stages of the fertility transition (which typically corresponds to more rural, preindustrial times).*

- *Almond, Douglas, and Janet Currie. “Killing Me Softly: The Fetal Origins Hypothesis.” Journal of Economic Perspectives 25, no. 3 (August 1, 2011): 153–72.* [*https://doi.org/10.1257/jep.25.3.153*](https://doi.org/10.1257/jep.25.3.153)*.*
- *Becker, Gary S., and H. Gregg Lewis. “On the Interaction between the Quantity and Quality of Children.” Journal of Political Economy 81, no. 2, Part 2 (1973): S279– 88. https://doi.org/10.1086/260166.*
- *Bongaarts, John. “Does Malnutrition Affect Fecundity? A Summary of Evidence.” Science 208, no. 4444 (1980): 564–69.*
- *Bongaarts, John. “Modeling the Fertility Impact of the Proximate Determinants: Time for a Tune-Up.” Demographic Research 33 (September 11, 2015): 535–60. https://doi.org/10.4054/DemRes.2015.33.19.*
- *Bongaarts, John, and Dennis Hodgson. Fertility Transition in the Developing World. SpringerBriefs in Population Studies. Cham: Springer International Publishing, 2022. https://doi.org/10.1007/978-3-031-11840-1.*
- *Friedlander, Dov. “Demographic Responses and Population Change.” Demography 6, no.*

*4 (November 1, 1969): 359–81.* [*https://doi.org/10.2307/2060083*](https://doi.org/10.2307/2060083)*.*

- *Lesthaeghe, Ron. “The Second Demographic Transition: A Concise Overview of Its Development.” Proceedings of the National Academy of Sciences 111, no. 51 (December 23, 2014): 18112–15. https://doi.org/10.1073/pnas.1420441111.*

Thank you for the recommended literature. During our study period, the population of Nouvelle France had not yet undergone the demographic transition, characterized by high birth and death rates [1]. However, we have incorporated discussions on demographic transitions into our manuscript narrative, emphasizing their relevance on lines 89–90 and in the lines 331–332 of the discussion.

*3. The authors make several normative statements regarding a younger age at first birth and*

*higher number of offspring being good (e.g., 175–176). While these may represent better reproductive performance in animal populations, it is well established in the human health literature that early age at first birth (often defined as 15 years old or younger) is associated with bad maternal and child health outcomes, such as low birthweight, preterm birth, infant mortality, and maternal mortality. In demographic literature, which emphasizes concepts of reproductive autonomy, it is not the total number of children born to a woman that matters but rather whether she is able to attain her desired family size. In many cases, her desired family size is lower than the number of children she has (so more is not unequivocally better), due to complex and intersecting social, cultural, and economic reasons. Beyond the issue with calling younger age at first birth and higher number of offspring “good”, this framing ignores (in the statistical sense) how these outcomes are in fact correlated, particularly for human populations, which are presumably quite different from red deer, elephants, and other animal populations in ways not acknowledged by the authors. As a result, certain findings are expressed as surprising, when in fact they are consistent with expectation for humans. For example, the authors state (374–379) that given the correlation between younger AFR and higher NO it was surprising that women born in urban (“bad”) parishes didn’t have more children surviving to adulthood (which they define as 15 years of age). Given younger AFR is associated with lower chances of child survival and a well-established reason for large numbers of pregnancies and children in preindustrial, pre-demographic transition times is to protect against child mortality and have a sufficiently large family to work in farms and other household activities.*

We absolutely agree with the reviewer, it has been shown that reproducing at a very young age (around or before age 15) is associated with greater mortality risks for the mother and the infant [18,19]. However, given our filtering approach (see 132–139) the population reproducing before 15 years old was marginal (0.21%) with not effect on the overall negative relationship between AFR and reproductive success. However, to address this comment, we did perform a sensitivity analysis that includes only the woman whose AFR is higher than 15 years old, which showed identical results. We now clarify this in lines 219–223. Also, in our population a higher number of offspring is a better outcome, given that the reproductive rate of a species evolves through natural selection, optimizing to support the highest number of individuals reaching maturity [20]. We elaborate on the correlation between humans and other mammals on the introduction, lines 41–62.

*Statistical Analyses:*

*1. While the procedure to remove insignificant terms from statistical models is appropriate*

*in some fields and for some analyses, in this case as the authors wish to test competing*

*hypotheses, any deletion of terms prevents their ability to do so in a systematic and*

*comprehensive manner. If the authors have a theory about which variables determine*

*these fertility outcomes, those variables should all be in the model regardless of statistical*

*significance because any lack of statistical significance informs the interpretation and*

*conclusion regarding the hypotheses.*

We appreciate the importance of testing competing hypotheses in a systematic and comprehensive manner. In response, we have updated our models to include all relevant variables, regardless of their statistical significance. As suggested, we have now also included the variable *Period* in the full models, as this aligns with our theoretical framework and enhances the robustness of our conclusions.

*2. To truly test these different hypotheses, I would suggest introducing the early life, later*

*life, and switching environmental variables in sequentially and then using all*

*combinations. This would allow the authors, for example, to see what happens to early*

*life environmental variables once they control for switching or once they control for later*

*life. Because this approach would also involve a large number of statistical tests, it would*

*be important for the authors to account for multiple hypotheses testing to ensure they are*

*not drawing conclusions from type I errors.*

Those models were in the first iterations of our analyses but faced a trade-off between testing many combinations of environments and statistical power. For instance, it is not statistically feasible to include the variables *Birth Environment, Adult Life Environment*, and their interaction in a single model. The reason for this is that there is only one urban parish in the north shore (Quebec City) and one urban parish in the south (Montreal), rendering singularities. Without this interaction, we believe it would be inaccurate to analyze the effects of birth and adult life environments independently. Therefore, we have decided to create a “Switch” variable to control for the adult life environment and the transition between early-life and adult-life environments. Furthermore, since urbanity and shore variables affect survival differently and the population distribution is irregular between the two urbanities but nearly 50% in each shore, we decided to divide them into “*Switching Shore*” and “*Switching Urbanity*.” We cannot include the A*dult Life Environment* variable alongside the *Switching Urbanity* or *Switching Shore* variables in the same model, because the “switch” already accounts for the environmental change. However, to further address the reviewer’s suggestion, we have sequentially added each fixed effect related to the environment (S6 Table) and constructed a table with all the models and their marginal and conditional R^2^, to allow the comparison between them (S7 Table). We clarify the matter in lines 245–249.

*3. A more minor comment on the interpretation of results is that the authors should be*

*careful in how they interpret terms on categorical variables and make sure they are*

*always interpreted as relative to the dropped category. For example, as stated in the notes*

*for Table 2 the reference level is rural south for birth environment, which means the*

*coefficients on rural north, urban south, and urban north are all interpreted as relative to*

*rural south. But in 216–218, the authors interpret the coefficients on the urban variables*

*and relative to both rural variables.*

Good point. This is why the comparison in lines 259–261, for example, uses the estimates shown in Fig 1A, which are based on the model’s predicted marginal values, obtained with the package *emmeans*. These estimates provide the value of each category independently and involve pairwise comparisons of the differences between all *Birth Environment* categories, rather than comparing each to a reference category [21]. For better clarification, the predicted marginal values described in the “Results” section and plotted in the figures have been included in the supplementary material as S8, S9, S10 and S11 Tables.

**BIBLIOGRAPHY**

1.	Gagnon A. Natural fertility and longevity. Fertility and Sterility. Elsevier Inc.; 2015. pp. 1109–1116. doi:10.1016/j.fertnstert.2015.03.030

2.	Bolund E. The challenge of measuring trade-offs in human life history research. Evolution and Human Behavior. 2020;41: 502–512. doi:10.1016/j.evolhumbehav.2020.09.003

3.	Lummaa V, Clutton-Brock T. Early development, survival and reproduction in humans. Trends Ecol Evol. 2002;17: 141–147. doi:10.1016/S0169-5347(01)02414-4

4.	Moore SE, Cole TJ, Poskitt EME, Sonko BJ, Whitehead RG, McGregor IA, et al. Season of birth predicts mortality in rural Gambia. Nature. 1997;388: 434–434. doi:10.1038/41245

5.	Hackman E, Emanuel I, Gerald Van Belle ;, Daling J. Maternal Birth Weight and Subsequent Pregnancy Outcome.

6.	Moore MP, Martin RA. On the evolution of carry‐over effects. Bouwhuis S, editor. Journal of Animal Ecology. 2019;88: 1832–1844. doi:10.1111/1365-2656.13081

7.	McMillen IC, Robinson JS. Developmental origins of the metabolic syndrome: Prediction, plasticity, and programming. Physiol Rev. 2005;85: 571–633. doi:10.1152/physrev.00053.2003

8.	Milot E, Mayer FM, Nussey DH, Boisvert M, Pelletier F, Reále D. Evidence for evolution in response to natural selection in a contemporary human population. Proc Natl Acad Sci U S A. 2011;108: 17040–17045. doi:10.1073/pnas.1104210108

9.	Voland E. Contributions of family reconstitution studies to evolutionary reproductive ecology. Evol Anthropol. 2000;9: 134–146. doi:10.1002/1520-6505(2000)9:3<134::AID-EVAN3>3.0.CO;2-M

10.	de Kat AC, Roelofs F, Slagboom PE, Broekmans FJ, Beekman M, van den Berg N. Late reproduction is associated with extended female survival but not with familial longevity. Reprod Biomed Online. 2024; 104073. doi:10.1016/j.rbmo.2024.104073

11.	Gagnon A. Effect of birth season on longevity: Thrifty and hopeful phenotypes in historical quebec. American Journal of Human Biology. 2012;24: 654–660. doi:10.1002/ajhb.22287

12.	Gagnon A, Bohnert N. Early life socioeconomic conditions in rural areas and old-age mortality in twentieth-century Quebec. Soc Sci Med. 2012;75: 1497–1504. doi:10.1016/j.socscimed.2012.06.007

13.	Dillon L. Parental and sibling influences on the timing of marriage, XVII th and XVIIIth century Québec. Ann Demogr Hist (Paris). 2010;119: 139–180. doi:10.3917/adh.119.0139

14.	Engelhardt S, Bergeron P, Gagnon A, Dillon L, Pelletier F. Using Geographic Distance as a Potential Proxy for Help in the Assessment of the Grandmother Hypothesis. Current Biology. 2019;29: 651-656.e3. doi:10.1016/j.cub.2019.01.027

15.	Gadoury L, Landry Y, Charbonneau H. Démographie différentielle en Nouvelle-France : villes et campagnes. Rev Hist Am Fr. 1985;38: 357. doi:10.7202/304283ar

16.	Pelletier F, Pigeon G, Bergeron P, Mayer FM, Boisvert M, Réale D, et al. Eco-evolutionary dynamics in a contemporary human population. Nat Commun. 2017;8: 1–5. doi:10.1038/ncomms15947

17.	Desjardins B. Bias in Age at Marriage in Family Reconstitutions: Evidence from French-Canadian Data. Popul Stud (NY). 1995;49: 165–169. doi:10.1080/0032472031000148306

18.	Gibbs CM, Wendt A, Peters S, Hogue CJ. The impact of early age at first childbirth on maternal and infant health. Paediatric and Perinatal Epidemiology. 2012. pp. 259–284. doi:10.1111/j.1365-3016.2012.01290.x

19.	Londero AP, Rossetti E, Pittini C, Cagnacci A, Driul L. Maternal age and the risk of adverse pregnancy outcomes: A retrospective cohort study. BMC Pregnancy Childbirth. 2019;19: 1–10. doi:10.1186/s12884-019-2400-x

20.	Lack D. The natural regulation of animal numbers. The Natural Regulation of Animal Numbers. 1954.

21.	Russell A, Lenth V, Buerkner P, Giné-vázquez I, Herve M, Love J, et al. Package ‘ emmeans ’ R topics documented : 2022. pp. 216–221.
